# Blood Pressure Lowering and Risk of Cancer: Individual Participant-Level Data Meta-Analysis and Mendelian Randomization Studies

**DOI:** 10.1016/j.jaccao.2025.03.005

**Published:** 2025-05-13

**Authors:** Milad Nazarzadeh, Emma Copland, Karl Smith Byrne, Dexter Canoy, Zeinab Bidel, Mark Woodward, Qianqian Yang, James McKay, Anders Mälarstig, Åsa K. Hedman, John Chalmers, Koon K. Teo, Carl J. Pepine, Barry R. Davis, Sverre E. Kjeldsen, Johan Sundström, Kazem Rahimi, Blood Pressure Lowering Treatment Trialists’ Collaboration

**Affiliations:** a Deep Medicine, Oxford Martin School, University of Oxford, Oxford, United Kingdom; b Nuffield Department of Women’s and Reproductive Health, University of Oxford, Oxford, United Kingdom; c National Institute for Health and Care Research Oxford Biomedical Research Centre, Oxford University Hospitals NHS Foundation Trust, Oxford, United Kingdom; d Cancer Epidemiology Unit, Nuffield Department of Population Health, University of Oxford, Oxford, United Kingdom; e Population Health Sciences Institute, Newcastle University, Newcastle upon Tyne, United Kingdom; f The George Institute for Global Health, University of New South Wales, Sydney, Australia; g The George Institute for Global Health, School of Public Health, Imperial College London, London, United Kingdom; h Genomic Epidemiology Branch, International Agency for Research on Cancer, Lyon, France; i Discovery Network, Pfizer Worldwide Research and Development, Stockholm, Sweden; j Department of Medicine, Karolinska Institute, Stockholm, Sweden; k Population Health Research Institute, Hamilton Health Sciences, McMaster University, Hamilton, Ontario, Canada; l College of Medicine, University of Florida, Gainesville, Florida, USA; m School of Public Health, The University of Texas, Houston, Texas, USA; n Department of Cardiology, University of Oslo, Ullevaal Hospital, Oslo, Norway; o Department of Medical Sciences, Clinical Epidemiology, Uppsala University, Uppsala, Sweden

**Keywords:** epidemiology, genetics, hypertension, ischemic disease, lifestyle risk factors, lung cancer, prevention, renal cell cancer

## Abstract

**Background:**

Pharmacologic blood pressure (BP) lowering is typically a lifelong treatment, and both clinicians and patients may have concerns about the long-term use of antihypertensive agents and the risk for cancer. However, evidence from randomized controlled trials (RCTs) regarding the effect of long-term pharmacologic BP lowering on the risk for new-onset cancer is limited, with most knowledge derived from observational studies.

**Objectives:**

The aim of this study was to assess whether long-term BP lowering affects the risk for new-onset cancer, cause-specific cancer death, and selected site-specific cancers.

**Methods:**

Individual-level data from 42 RCTs were pooled using a one-stage individual participant data meta-analysis. The primary outcome was incident cancer of all types, and secondary outcomes were cause-specific cancer death and selected site-specific cancers. Prespecified subgroup analyses were conducted to assess the heterogeneity of the BP-lowering effect by baseline variables and over follow-up time. Cox proportional hazards regression, stratified by trial, was used for the statistical analysis. For site-specific cancers, analyses were complemented with Mendelian randomization, using naturally randomized genetic variants associated with BP lowering to mimic the design of a long-term RCT.

**Results:**

Data from 314,016 randomly allocated participants without known cancer at baseline were analyzed. Over a median follow-up of 4 years (Q1-Q3: 3-5 years), 17,954 participants (5.7%) developed cancer, and 4,878 (1.5%) died of cancer. In the individual participant data meta-analysis, no associations were found between reductions in systolic or diastolic BP and cancer risk (HR per 5 mm Hg reduction in systolic BP: 1.03 [95% CI: 0.99-1.06]; HR per 3 mm Hg reduction in diastolic BP: 1.03 [95% CI: 0.98-1.07]). No changes in relative risk for incident cancer were observed over follow-up time, nor was there evidence of heterogeneity in treatment effects across baseline subgroups. No effect on cause-specific cancer death was found. For site-specific cancers, no evidence of an effect was observed, except a possible link with lung cancer risk (HR for systolic BP reduction: 1.17; 99.5% CI: 1.02-1.32). Mendelian randomization studies showed no association between systolic or diastolic BP reduction and site-specific cancers, including overall lung cancer and its subtypes.

**Conclusions:**

Randomized data analysis provided no evidence to indicate that pharmacologic BP lowering has a substantial impact, either increasing or decreasing, on the risk for incident cancer, cause-specific cancer death, or selected site-specific cancers.

Hypertension, which requires long-term pharmacologic blood pressure–lowering treatment, is the most commonly observed coexisting condition in patients with cancer.^1^ There has been a long-standing debate about the effect of antihypertensive drugs on cancer risk, but a large-scale meta-analysis of individual participant data (IPD) has largely dismissed any detrimental impacts of antihypertensive drug classes on the overall risk for cancer, as well as on the incidence of various site-specific cancer types.^2^ Nevertheless, the examination of the direct effects attributable to blood pressure reduction itself remains inadequately investigated, with the majority of evidence deriving from observational studies.^3^, ^4^, ^5^, ^6^

High blood pressure and cancer share common risk factors and biological pathways, including age, unhealthy lifestyle, smoking, body mass index,^3^^,^^4^^,^^7^^,^^8^ and immune system dysfunction.^9^^,^^10^ This intricate relationship has led to difficulties in evaluating any potential causal association between blood pressure, pharmacologic blood pressure lowering and cancer risk.^11^ This challenge is further compounded by the necessity to discern whether the associations previously identified in extant literature are indeed reflective of a direct causative relationship or if they are merely the result of residual confounding or instances of reverse causality.^3^ Such separation is crucial for formulating effective and safe long-term blood pressure–lowering treatment strategies.

Randomized evidence regarding the effect of blood pressure reduction per se on cancer risk is constrained by several key factors: insufficient sample sizes and event rates in individual randomized controlled trials (RCTs), which are not often designed to capture cancer as a primary or secondary outcome or even as a severe adverse event. Additionally, the typically short duration of follow-up in RCTs might be inadequate to assess long-term effects, particularly for outcomes such as cancer, which have long induction periods. Furthermore, the heterogeneity of interventions for blood pressure reduction, encompassing various medications, complicates the isolation of their specific effects on cancer risk, adding to the complexity of interpreting individual trial results. Consequently, relying solely on data from a single RCT, or from an observational study, might poses limitations when attempting to address the aforementioned knowledge gap and provide compelling randomized evidence.

Therefore, we used a strategy of evidence triangulation.^12^ We conducted an IPD meta-analysis of RCTs in conjunction with a Mendelian randomization study using genetic data as an independent source of randomized evidence. This dual-study approach not only enhances the robustness of our findings but also ensures a wide-angle understanding of the relationship between blood pressure modulation and cancer risk.

## Methods

We implemented 2 distinct study designs, each leveraging independent sources of randomized data. First, we conducted a meta-analysis of IPD from RCTs of pharmacologic blood pressure–lowering interventions to examine the effect of systolic and diastolic blood pressure reduction on the risk for outcomes among participants randomly allocated to intervention and comparator arms. Second, we performed a Mendelian randomization analysis using naturally randomized genetic variants associated with systolic and diastolic blood pressure, thereby emulating the random allocation principle of RCTs, to evaluate the long-term effects of blood pressure reduction on the risk for site-specific outcomes.^13^

### Individual Participant Data Meta-Analyses of RCTS

#### Study setting, design, and eligibility criteria

This IPD meta-analysis was based on the resource provided by the Blood Pressure Lowering Treatment Trialists’ Collaboration (BPLTTC),^14^^,^^15^ an international collaboration of the principal investigators of major clinical trials of pharmacologic blood pressure–lowering treatments. Further details of the current cycle of BPLTTC activities, the search strategy, and inclusion criteria have been published previously.^14^ The collaboration currently has access to IPD from 52 RCTs involving 363,684 participants. Eligible trials were those with at least 1,000 participant-years of follow-up in each trial arm and individual-level information on cancer events, timing of diagnosis, and duration of follow-up for those without cancer diagnoses. Ethics approval for the current cycle was obtained from the Oxford Central Ethics University Research Committee (545-14). The present study protocol was approved by the steering committee and collaborators before releasing the data for analysis.

#### Treatment and comparator arms

Each participant was assigned to a treatment or comparator arm on the basis of their random allocation in individual trials according to the trial design. Details of the assignment of treatment and comparator groups for each trial have been described previously.^15^ Briefly, in placebo-controlled trials, those randomly allocated to the active group were assigned to the treatment arm, and those in the placebo group were assigned to the comparator arm; in trials comparing blood pressure–lowering intensities, a lower blood pressure target was considered the treatment and a higher blood pressure target the comparator; and in head-to-head trials comparing various drug classes, the drug class with the greater blood pressure reduction was assigned as the treatment arm and the other drug class(es) as the comparator arm.

#### Primary and secondary outcomes

The primary outcome of this study was any incident cancer, defined as the first report of a cancer diagnosis after randomization. The secondary outcomes encompassed cause-specific cancer death and various site-specific cancers for which event data were available in the BPLTTC data set. These included cancers of the breast, colorectal region, kidney, lung, prostate, and skin. Outcomes were defined on the basis of the diagnostic and adjudication criteria of each trial.

#### Statistical analysis

The characteristics of participants assigned to the treatment and comparator arms at baseline are presented as mean ± SD or as median (Q1-Q3) for continuous variables and as count (percentage) for categorical variables. The IPD meta-analysis was conducted using the 1-stage approach, in which data from all trials are analyzed simultaneously.^16^ HRs with 95% CIs were estimated using fixed-effects Cox proportional hazards models, stratified by trial, and were based on the intention-to-treat principle. The timing of the event was defined as the date of cancer diagnosis in trials in which this was reported; otherwise, it was defined as the date the cancer was reported in the trial or the date of death if cancer was recorded only as an underlying cause of death. Individuals were censored at the date of death, last follow-up visit, or administrative censoring at the end of the trial. The effect sizes were standardized for a 5 mm Hg reduction in systolic blood pressure and a 3 mm Hg reduction in diastolic blood pressure between randomized groups to account for heterogeneity in blood pressure reduction across different trials.^16^ These values represent the round values closest to the weighted means of systolic and diastolic blood pressure reductions across all trials, excluding head-to-head trials^15^ (Supplemental Method 1).

We assessed temporal variation in relative risk by estimating the HRs for specific time periods during follow-up. In this analysis, patients contributed to the exposure time during each time period until they either developed the outcome or were censored. We conducted subgroup analyses on the basis of baseline patient characteristics, including systolic and diastolic blood pressure levels, age, sex, body mass index, smoking status, and previous antihypertensive drug use, incorporating an interaction term between each characteristic and the treatment arm in the model. The heterogeneity of treatment effect across subgroups and time periods was assessed using likelihood ratio tests, adjusted for multiple comparisons.^17^^,^^18^ In a sensitivity analysis, we used Fine and Gray subdistribution hazard models to account for the competing risk for any noncancer death.

Analyses were conducted separately for each site-specific cancer, and the results were compared with findings from Mendelian randomization (details described later). In this analysis, which examined 6 site-specific cancers using 2 study designs simultaneously, we report effect sizes with 99.5% CIs to reduce the risk for false-positive results.^19^

### Mendelian randomization

We used a 2-sample Mendelian randomization framework to assess the effect of blood pressure lowering on secondary outcomes and compared the results with the findings from the IPD meta-analysis. Within this framework, blood pressure–lowering randomized treatment was mimicked using naturally and randomly allocated genetic variants.^13^^,^^20^ The exposure was genetically determined midlife systolic and diastolic blood pressure that was estimated using genetic variants with minor allele frequencies >0.01 that were independently (linkage disequilibrium *r*^2^ < 0.05) associated with blood pressure at a genome-wide significance level (*P* < 5 × 10^−8^).^21^ The summary statistics for variants associated with each site-specific cancer as secondary outcomes were extracted from relevant genome-wide association studies (GWAS). Details of GWAS used for this analysis are described in Supplemental Method 2. On the basis of the information provided in each GWAS report, there was no known overlap between the GWAS data used for exposures and outcomes.

The summary estimates of variants-exposure and variants-outcome were harmonized before conducting the statistical analysis.^22^^,^^23^ The inverse variance weighted method was used as the primary method. As this assumes that either all the instruments are valid or any horizontal pleiotropy is balanced,^24^ we applied various other Mendelian randomization methods with different assumptions as sensitivity analyses. We used the weighted median method,^25^ which is consistent if at least 50% of the weight comes from valid instrumental variables.^26^ The Mendelian randomization pleiotropy residual sum and outlier method was used to test and, if needed, correct for any possible horizontal pleiotropic outliers in the analysis.^27^ The Mendelian randomization Egger regression method was used to assess the presence of pleiotropy.^28^ The robust adjusted profile score method is robust to systematic and idiosyncratic pleiotropy and is recommended for complex traits and diseases.^29^ The Mendelian randomization mix method provides unbiased estimation in the presence of a large number of invalid genetic instruments.^30^ Furthermore, we examined the heterogeneity of the estimates by using the Cochran *Q* test.^31^ All statistical analyses were performed using R version 3.3 (R Foundation for Statistical Computing).

## Results

Of the 52 trials in the BPLTTC database, 10 trials did not report on cancer outcomes and were therefore excluded, leaving a total of 42 trials involving 314,016 individuals for inclusion in this analysis. The general characteristics of each trial, including the source of outcome ascertainment and the level of detail provided for cancer outcomes, are presented in Supplemental Method 3. Forty-one percent of the participants were women, and the mean age was 66 ± 6 years. Additional participant baseline characteristics are detailed in Table 1, and information on individual trials has been published previously.^15^^,^^16^ Over a median of 4 years (Q1-Q3: 3-5 years), 17,954 participants were diagnosed with cancer of any type, and 4,878 participants were reported to have died with cancer as the cause of death.

**Table 1:** Baseline Characteristics of Participants Included in the 1-Stage Individual Participant Data Meta-Analysis

	Treatment (n = 144,997)	Comparator (n = 169,019)
Sex		
Female	59,711 (41)	69,574 (41)
Male	85,286 (59)	99,445 (59)
Age, y	65 ± 9	66 ± 9
Age categories		
<65 y	65,779 (45)	73,535 (44)
≥65 y	79,202 (55)	95,455 (56)
Systolic blood pressure, mm Hg	151 ± 21	151 ± 21
Diastolic blood pressure, mm Hg	86 ± 12	86 ± 12
Systolic blood pressure categories		
<130 mm Hg	20,090 (14)	24,081 (13)
130-139 mm Hg	21,019 (15)	24,647 (15)
140-149 mm Hg	28,624 (20)	33,495 (20)
150-159 mm Hg	24,976 (17)	29,493 (17)
≥160 mm Hg	49,928 (34)	56,927 (35)
Diastolic blood pressure categories		
<70 mm Hg	10,101 (7)	12,071 (7)
70-79 mm Hg	27,016 (19)	32,542 (19)
80-89 mm Hg	47,499 (33)	55,655 (33)
90-99 mm Hg	40,418 (28)	46,620 (28)
≥100 mm Hg	19,573 (14)	21,734 (13)
Body mass index, kg/m^2^	28 ± 5	28 ± 5
Body mass index categories		
<25 kg/m^2^	34,679 (29)	39,924 (28)
25-29 kg/m^2^	50,131 (42)	60,697 (42)
≥30 kg/m^2^	34,387 (29)	42,313 (30)
Current smoking	27,329 (19)	30,971 (18)
Previously on blood pressure–lowering medication	84,633 (70)	104,888 (74)
Follow-up duration, y, median (interquartile range)	4.2 (2.0)	4.3 (1.9)

Values are n (%) or mean ± SD unless otherwise indicated.

**Figure 1:**
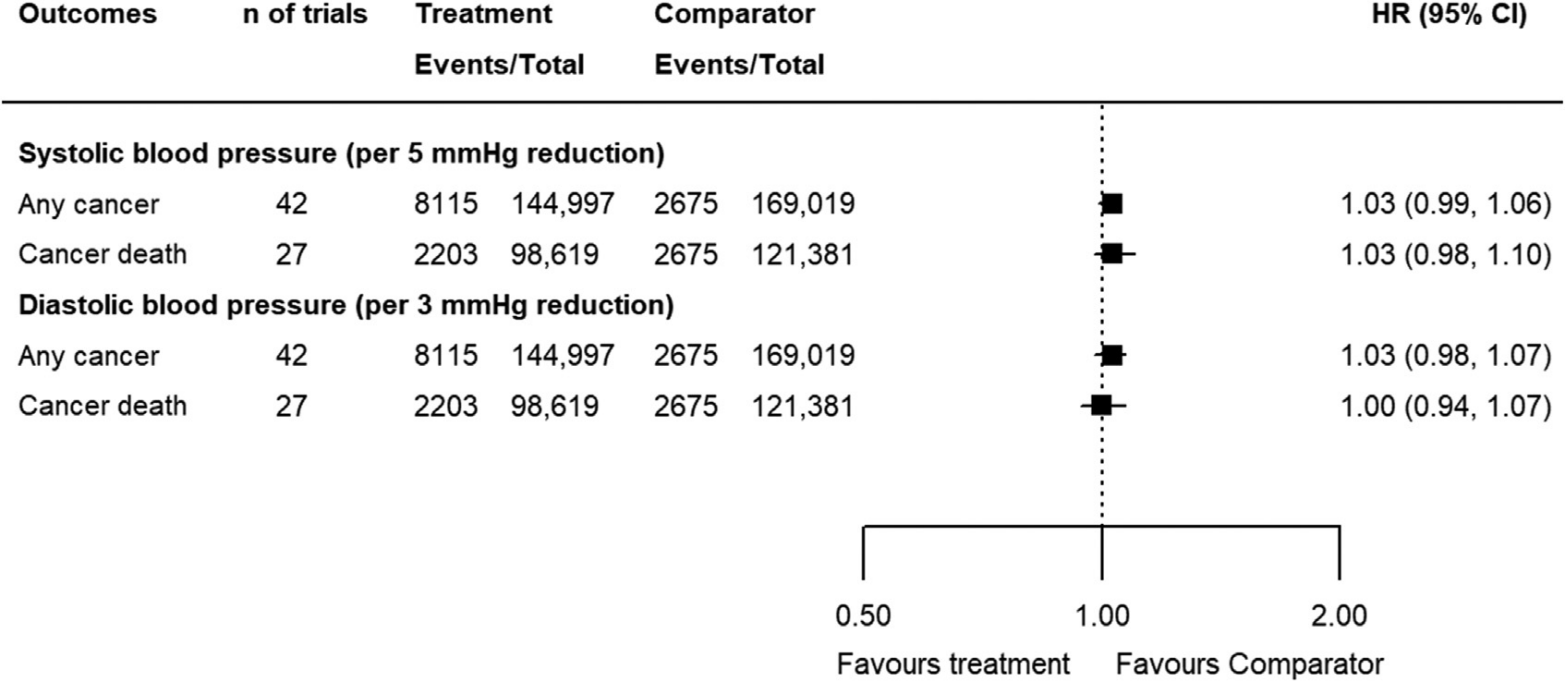
Blood Pressure–Lowering Treatment and Risk for Incident Cancer and Cancer Deaths Effect of blood pressure–lowering treatment on the risk for incident cancer and cancer deaths. HRs with 95% CIs are presented for reductions in systolic blood pressure (5 mm Hg) and diastolic blood pressure (3 mm Hg). Data are pooled from 42 trials for any cancer incidence and 27 trials for cancer mortality, comparing treatment vs comparator groups. The HRs suggest no significant association between blood pressure reduction and cancer risk or death. The forest plot visually represents effect estimates, with the dotted line at an HR of 1, indicating no effect. A 95% CI crossing 1 suggests nonsignificant differences between groups.

**Figure 2:**
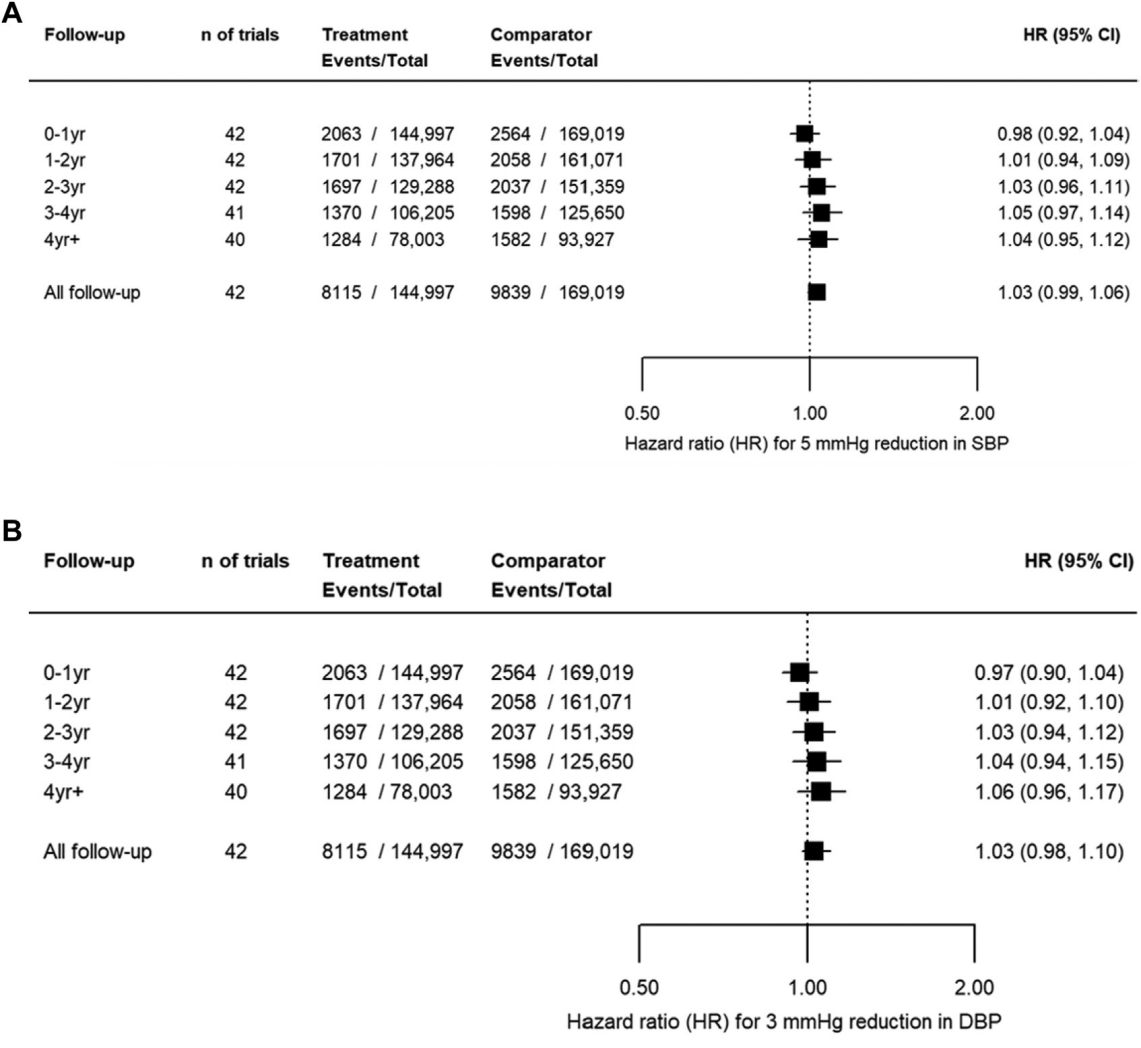
Blood Pressure–Lowering Treatment and Risk for Incident Cancer, Stratified by Follow-Up Time Blood pressure–lowering treatment and the risk for incident cancer, stratified by follow-up duration. (A) HRs with 95% CIs for a 5 mm Hg reduction in systolic blood pressure (SBP) across different follow-up periods. (B) HRs for a 3 mm Hg reduction in diastolic blood pressure (DBP). Data are pooled from 42 trials, with HRs estimated for follow-up durations ranging from 0 to 1 year to ≥4 years. The results indicate no significant association between blood pressure reduction and cancer risk over time, with all CIs crossing unity. Forest plots illustrate effect estimates.

**Figure 3:**
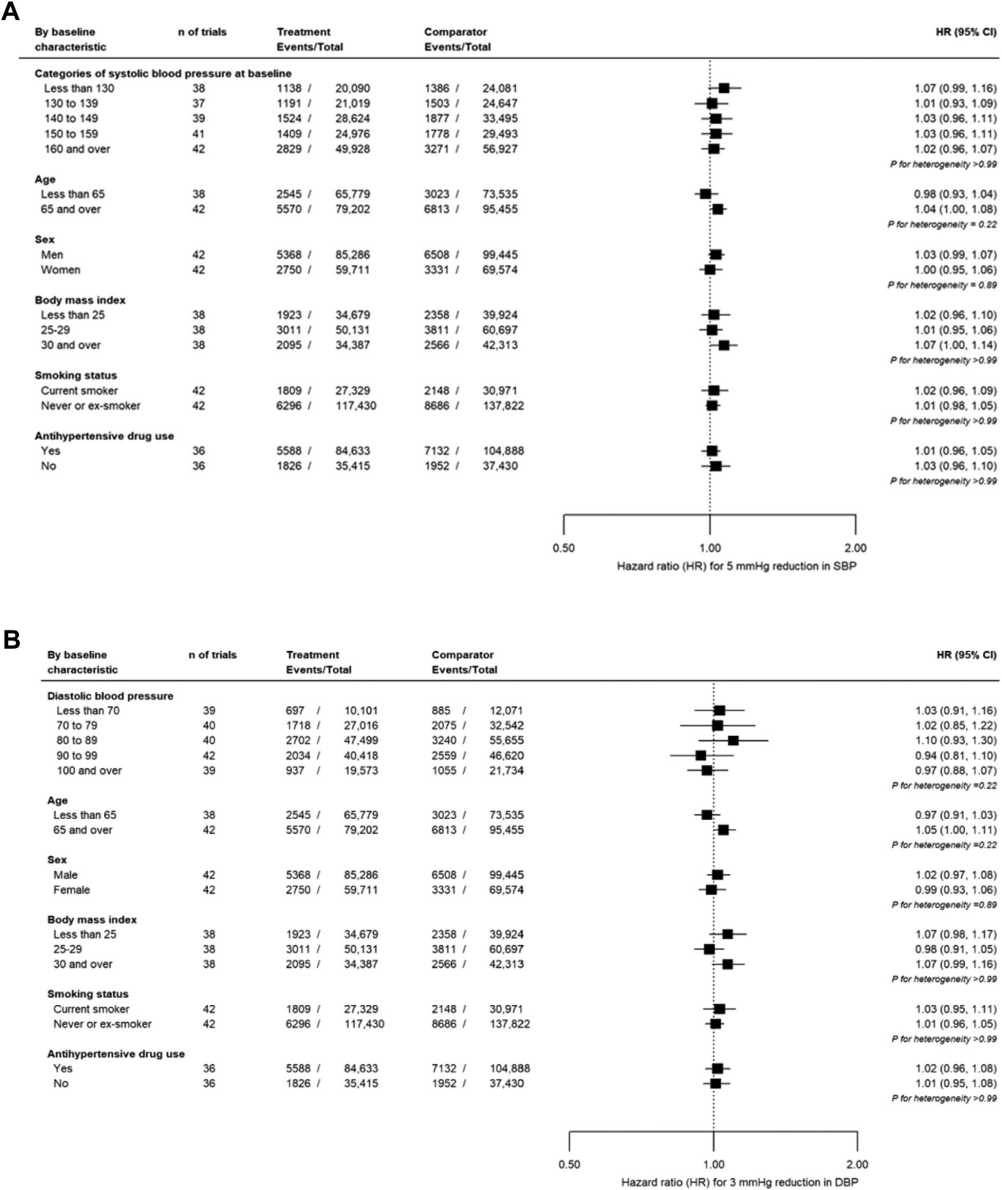
Effects of Blood Pressure Lowering on Incident Cancer Stratified by Baseline Characteristics of Participants Effects of blood pressure–lowering treatment on the risk for incident cancer, stratified by baseline participant characteristics. (A) HRs with 95% CIs for a 5 mm Hg reduction in SBP. (B) HRs for a 3 mm Hg reduction in DBP. Data are stratified by baseline blood pressure, age, sex, body mass index, smoking status, and antihypertensive drug use across 42 trials. No significant associations were observed across subgroups, with all CIs crossing unity. Forest plots display effect estimates, and *P* values for heterogeneity assess subgroup differences. Abbreviations as in Figure 2.

The relative risk for diagnosis of incident cancer of any type for a 5 mm Hg reduction in systolic blood pressure was 1.03 (95% CI: 0.99-1.06) and for a 3 mm Hg reduction in diastolic blood pressure was 1.03 (95% CI: 0.98-1.07) (Figure 1). For the outcome of cancer death, the corresponding HRs for systolic and diastolic blood pressure were 1.03 (95% CI: 0.98-1.10) and 1.00 (95% CI: 0.94-1.07), respectively. In the analysis that investigated temporal variation in relative risk throughout the follow-up period, no evidence was found indicating patterns of increasing or decreasing risk over time for any cancer (Figure 2). No evidence of heterogeneity in the relative risk for any cancer was observed between men and women, among different age groups, among various baseline blood pressure levels, among body mass index categories, by smoking status, or by history of blood pressure–lowering treatment prior to randomization (*P* for interaction >0.22 for all) (Figure 3). The HRs estimated from the Fine and Gray subdistribution models were comparable with the HRs from the primary analysis, suggesting that there was no bias due to competing risks (Supplemental Figure 1).

**Figure 4:**
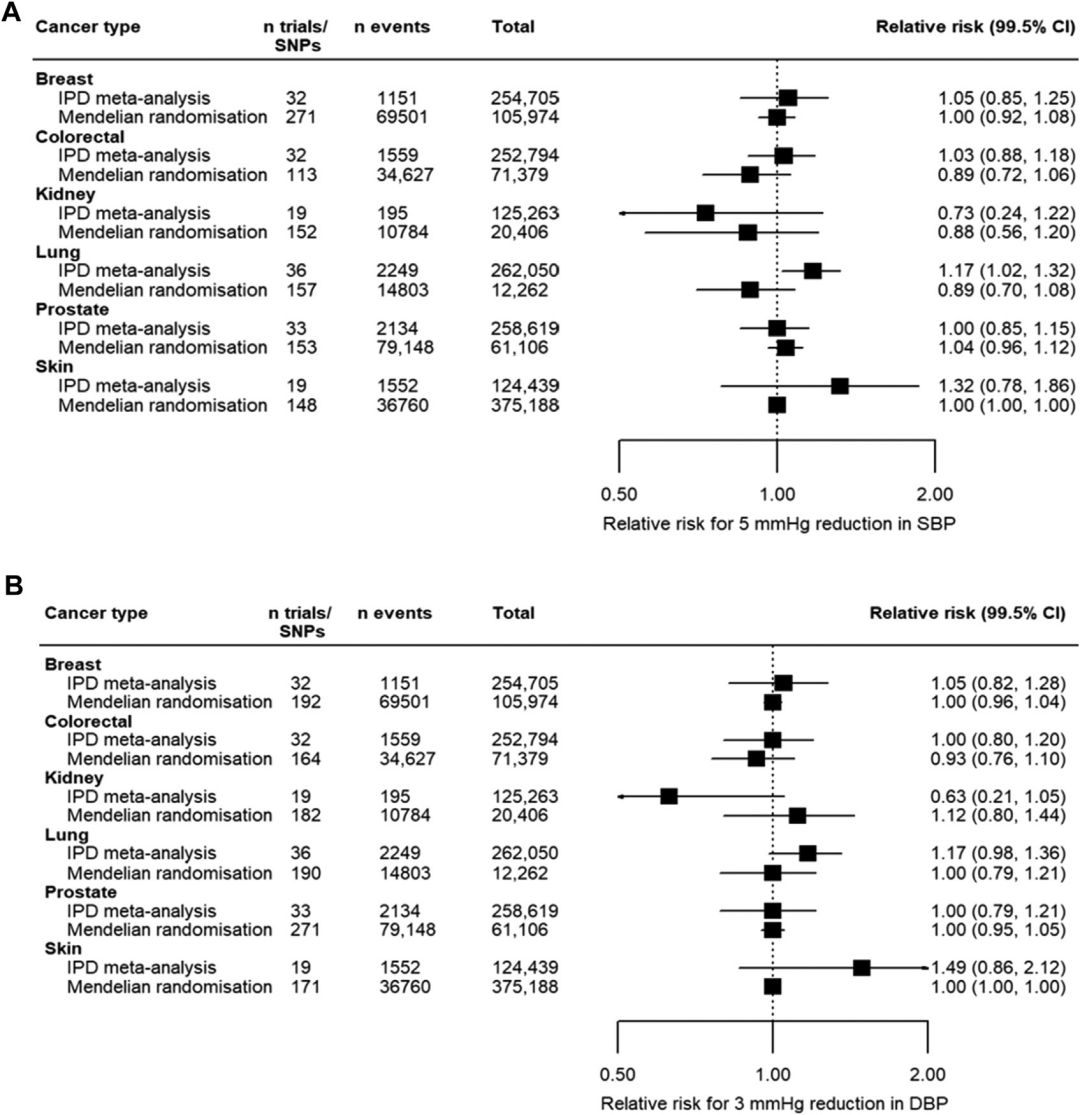
Effects of Blood Pressure Lowering on Site-Specific Cancer, Separately Estimated Using Individual Participant Data Meta-Analysis and a Mendelian Randomization Study Effects of blood pressure–lowering treatment on site-specific cancer risk, separately estimated using individual participant data (IPD) meta-analysis and Mendelian randomization. (A) RRs with 99.5% CIs for a 5 mm Hg reduction in SBP. (B) RRs for a 3 mm Hg reduction in DBP. Cancer risk estimates are provided for breast, colorectal, kidney, lung, prostate, and skin cancers. Findings using both analytical approaches suggest no consistent associations between blood pressure reduction and site-specific cancer risk, with all CIs crossing unity. Forest plots illustrate effect estimates. SNP = single-nucleotide polymorphism; other abbreviations as in Figure 2.

In the site-specific cancer analysis, no effect was found between the reduction of systolic or diastolic blood pressure and the risk for developing breast, colorectal, kidney, prostate, and skin cancers (Figure 4). An increased risk for overall lung cancer was observed with the reduction of systolic blood pressure (HR: 1.17; 99.5% CI: 1.02-1.32), accompanied by a marginal increase in risk for diastolic blood pressure reduction (HR: 1.17; 99.5% CI: 0.98-1.36) (Figure 4).

For site-specific cancers, the results from the Fine and Gray subdistribution models were comparable with those of the primary analysis, suggesting no competing risk effects (Supplemental Figure 1). The results of the post hoc power calculation are reported in Supplemental Result 1. The findings confirm that we had at least 80% statistical power to detect a 3% change in effect size for both cancer incidence and cause-specific cancer death. For site-specific cancers, the analysis demonstrated that for cancers with more than 1,000 events, there was 80% power to detect a relative risk reduction of at least 7%.

**Figure 5:**
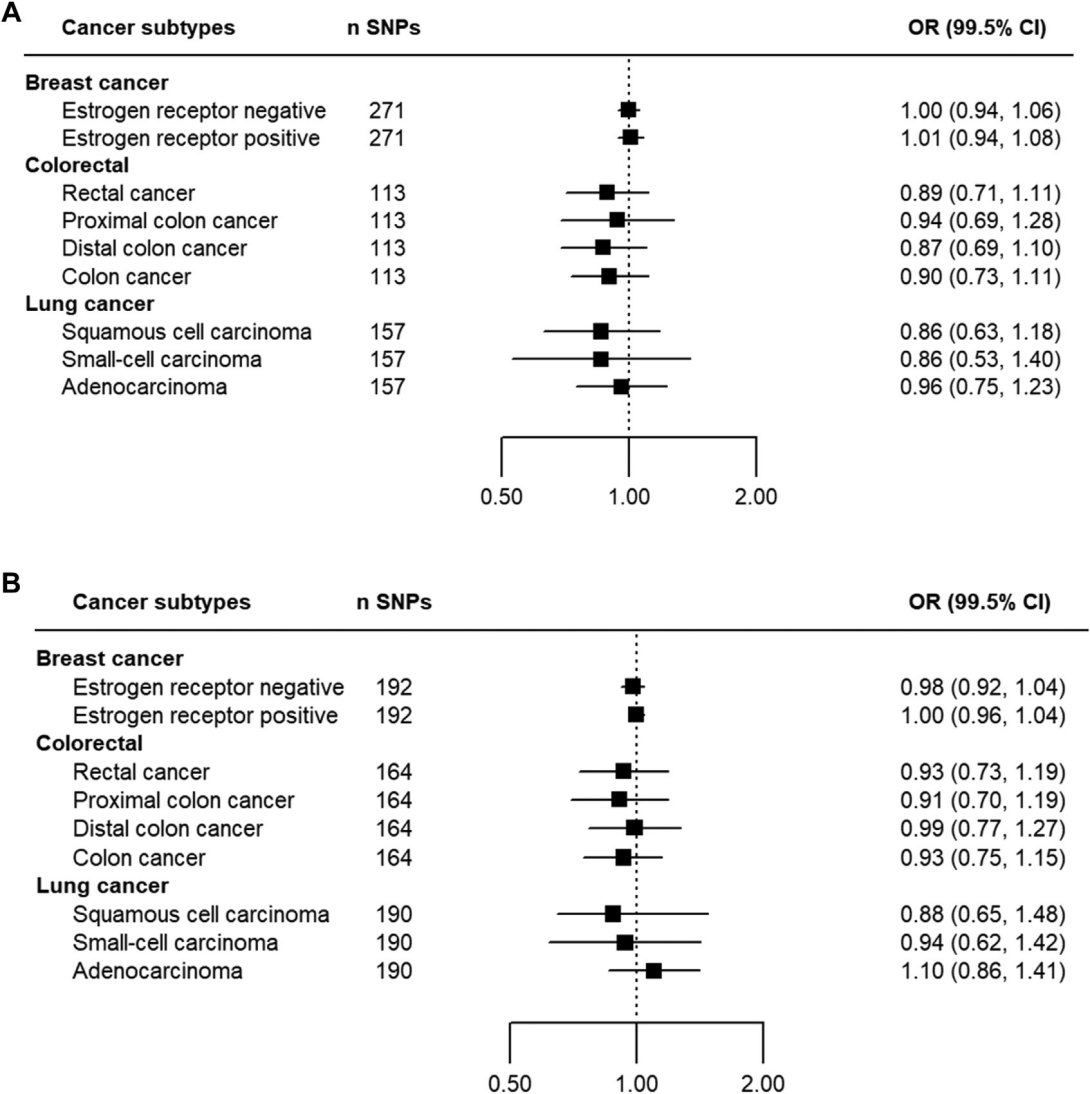
Mendelian Randomization Analysis of the Effects of Blood Pressure Lowering on Breast, Colorectal, and Lung Cancer Subtypes (A) ORs with 99.5% CIs for a 5 mm Hg reduction in SBP. (B) ORs for a 3 mm Hg reduction in DBP. Subgroup analyses include estrogen receptor–positive and estrogen receptor–negative breast cancer, rectal and colon cancer subtypes, and lung cancer histologic subtypes. The n SNPs column indicates the number of SNPs used as instrumental variables for genetic proxies of blood pressure. Findings indicate no significant associations between blood pressure reduction and cancer risk across subtypes, with all CIs crossing unity. Forest plots illustrate effect estimates. Abbreviations as in Figures 2 and 4.

In Mendelian randomization analyses for each site-specific cancer, we found no evidence that each 5 mm Hg genetically influenced lower systolic blood pressure reduction or 3 mm Hg diastolic blood pressure reduction was associated with any of the cancers investigated. A comparison between the IPD meta-analysis and Mendelian randomization estimations is presented in Figure 4. In more detailed analyses for cancer subtypes, no material effect was found for subtypes of colorectal cancers (colon, distal colon, proximal colon, and rectal), lung cancers (squamous cell carcinoma, small cell carcinoma, and adenocarcinoma) and breast cancers (estrogen receptor positive and negative) (Figure 5). No material change was found in sensitivity analyses using different methods of Mendelian randomization (Supplemental Results 2-5).

## Discussion

In the IPD meta-analysis of major blood pressure–lowering trials, our findings indicate no association between pharmacologic blood pressure reduction and the incidence of cancer or cancer-related death over a median follow-up period of 4 years after treatment initiation. Time-stratified analysis further demonstrated a consistent null relative risk for total cancer incidence throughout the median 4-year follow-up duration. Detailed investigations into site-specific cancers also did not reveal substantial evidence of impact on the risk for breast, colorectal, kidney, prostate, and skin cancers. However, the possibility of an elevated risk for overall lung cancer in relation to blood pressure reduction cannot be conclusively dismissed. Complementary to these findings, genetic analyses using Mendelian randomization as an independent source of randomized data did not support any relationship between blood pressure reduction and the risk for any site-specific cancer types addressed in the IPD meta-analysis, including a null effect for overall lung cancer and its subtypes (Central Illustration).

**Central Illustration:**
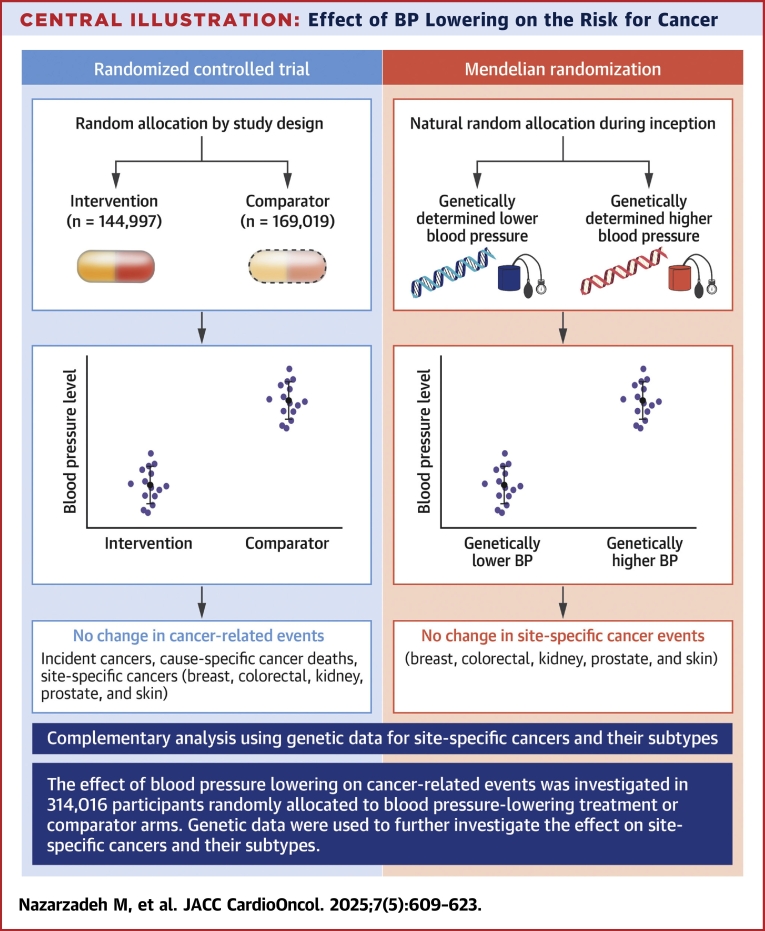
Effect of BP Lowering on the Risk for Cancer The effect of blood pressure (BP) lowing on cancer-related events was investigated in 314,016 participants randomly allocated to BP-lowering treatment or comparator arms. Genetic data were then used to further investigate the effect on site-specific cancers.

The majority of evidence regarding the association between blood pressure and cancer development originates from observational population-based data. A meta-analysis incorporating observational data from 85 prospective studies indicated that hypertension leads to an elevated risk for developing kidney, colorectal, and breast cancers, whereas it shows no significant effect on the incidence of lung and prostate cancers.^3^ Overall, the most consistent evidence from observational studies was observed for kidney cancer^5^^,^^32^^,^^33^ and breast cancer,^3^^,^^7^ for which several reports indicated a higher risk associated with hypertension or elevated blood pressure. Similarly, observational studies predominantly indicate a positive association between elevated blood pressure and cancer death or survival.^34^, ^35^, ^36^ Despite the overall observational data hinting at a link between high blood pressure and increased risk for cancer occurrence, cancer-related death, and several site-specific cancers, the inherent nature of this evidence, coupled with limitations such as treating continuous blood pressure measurement as a binary biomarker, small number of cancer events, risk for false-positive associations when investigating multiple cancers concurrently, and possible residual confounding and reverse causation, collectively hinders the ability to draw strong clinical implications. To address the limitation of relying on observational evidence, several Mendelian randomization studies have been previously conducted. Analyses of the large-scale China Kadoorie Biobank showed that higher levels of systolic blood pressure were unrelated to the risk for overall incident cancer.^37^ Another study, which adjusted for multiple testing, revealed no association between systolic and diastolic blood pressure and the risk for total or site-specific cancer, covering all cancer types included in our study.^38^ In contrast, a Mendelian randomization study focusing on the association between blood pressure and the risk for renal cell carcinoma revealed evidence of a positive association, specifically identifying a link between increased diastolic blood pressure and a higher risk for developing renal cell carcinoma.^39^ These findings collectively highlight the complexity of the relationship between blood pressure and cancer risk, indicating the need for further randomized evidence to fully comprehend these associations and their implications in clinical practice.

There is a lack of existing evidence from RCTs investigating the effect of pharmacologic blood pressure–lowering per se on incident cancer. Previous studies have mainly examined the safety of specific antihypertensive drug classes in the context of cancer risk, but they did not explore the effect of a unit reduction in blood pressure achieved through taking these medications.^2^^,^^40^ Individual RCTs often lack the necessary statistical power to provide robust evidence for this effect, attributable to the small number of event occurrences and the absence of long-term follow-up. Our study fills this gap in evidence by pooling individual-level data from randomized trials of blood pressure–lowering treatment. We have shown that blood pressure lowering does not have an effect on the risk for incident cancer or cancer death, with no increase or decrease during follow-up time. The absence of temporal change in relative risk is noteworthy, as it serves as an indicator of the lack of a causal effect, especially within the context of data derived from RCTs.

Our analysis of data from RCTs suggested an increased risk for overall lung cancer. Previous aggregate data meta-analyses of RCTs have highlighted a potential augmentation in lung cancer risk associated with angiotensin receptor blockers.^41^^,^^42^ In contrast, earlier IPD meta-analysis from BPLTTC have not substantiated an impact of angiotensin receptor blockers or any other major class of antihypertensive drugs on the risk for lung cancer.^2^ There are certain aspects to consider when interpreting RCT data in context of cancer. Trials have a comparably short follow-up time, which may not be sufficient to enable us to attribute the onset of lung cancer to pharmacologic blood pressure lowering. The latency period for solid tumors such as lung cancer tends to be long. For example, the average latency interval between the onset of a well-known carcinogen such as smoking and the diagnosis of lung cancer is approximately 50 years.^43^ Given this limitation, Mendelian randomization was used for a more thorough examination. This analysis revealed no significant association between the reduction of blood pressure and particular subtypes of lung cancer, encompassing adenocarcinoma, small cell, and squamous cell carcinoma. In contrast to RCTs, Mendelian randomization uses genetic variants as proxies for lifelong exposure to blood pressure levels, offering a potentially more accurate picture of long-term effects, which is crucial when dealing with a disease that has a long latency period.^44^ This method leverages the random allocation of genes at conception, akin to the randomization in controlled trials, to minimize confounding effects. Taking into account the entirety of randomized evidence, including both RCTs and Mendelian randomization, and considering the limitations and strengths of each study design, we conclude that there is no convincing evidence to support a link between blood pressure lowering and the development of lung cancer.

From a biological perspective, elevated blood pressure and hypertension have been implicated in various metabolic pathways that may contribute to carcinogenesis. For instance, hypertension is associated with increased lipid peroxidation, which has been proposed as a mechanism responsible for a higher risk for renal cell carcinoma.^45^ Additionally, activation of the renin-angiotensin-aldosterone system in individuals with hypertension has been implicated in processes such as cellular proliferation, inflammation, angiogenesis, and tissue remodeling, potentially increasing cancer risk.^46^ Moreover, elevated blood pressure can lead to increased levels of endothelin, a potent vasoconstrictor, which, when overexpressed, has been associated with several types of cancer.^47^ Despite these mechanistic associations, we found no clinical evidence for the effect of blood pressure lowering on cancer risk. This suggests that although hypertension may be a marker for increased cancer risk in experimental and observational studies, particularly given significant shared risk factors, the clinical reduction of blood pressure through antihypertensive therapy does not appear to significantly alter cancer incidence.

The key strength of this study lies in its novelty and in its large sample size, leveraging individual-level data from pharmacologic blood pressure–lowering RCTs to evaluate the effect of unit decreases in blood pressure on cancer risk. This allowed us to investigate whether cancer risk can be modified through blood pressure lowering, both overall and within subgroups defined by participant baseline characteristics, including important risk factors shared by both conditions. The ability to conduct time-stratified analyses was also an important strength of the study to account for any potential latency period between initiation of blood pressure–lowering treatment and tumor formation.^2^ Additionally, using 2 study designs enabled us to validate our findings through independent sources of randomized data, thereby addressing some of the limitations inherent in each individual methodology.

### Study limitations

One limitation of the IPD meta-analysis is that we did not have access to IPD from all eligible trials. Although we had sufficient statistical power for both primary and secondary outcomes, the analysis for kidney cancer, with only 195 events, was underpowered and yielded wide CIs. However, we were able to triangulate these findings using the results from the Mendelian randomization analysis, which were based on genome-wide association analyses of a much larger number of cancer events than available in the BPLTTC database, and therefore generally had narrower CIs.

Another limitation was the relatively short median follow-up duration of 4 years across the pooled trials, which may not be long enough for cancers to develop after blood pressure lowering is initiated. We conducted time-stratified analyses to address this limitation, as we assumed that a pattern of increasing or decreasing risk over time would indicate an emerging harmful or protective effect in later years. Furthermore, Mendelian randomization models the effects of lifetime blood pressure reduction, directly addressing this limitation in the evidence from RCTs. Additionally, cancer was not the primary outcome in any of the trials, so case ascertainment and adjudication were not consistent across trials. However, this is unlikely to introduce bias in randomized trials as any event misclassification is expected to be consistent across treatment groups.^48^^,^^49^

There was also uncertainty around whether cancers in some trials were incident diagnoses, because of incomplete data on the history of cancer at baseline. However, our previous work on this data set suggested that stratification by explicit exclusion of cancer patients at baseline does not lead to any differences in relative treatment effects.^2^

Finally, we did not examine the class-specific effects of antihypertensive medications on the risk for each site-specific cancer and their subtypes. As each drug class may act through different pathways (targeted or known and unknown off targets), investigating all potential mechanisms within a single study is challenging and requires predefined hypotheses for specific pathways. Therefore, assessing the effects of specific antihypertensive drug classes, such as the impact of renin-angiotensin system inhibitors on colorectal cancer observed in previous studies,^50^ warrants further investigation.

## Conclusions

This large-scale study of individual-level data from randomized trials has provided evidence that pharmacologic blood pressure lowering per se is not associated with an increased or decreased risk for being diagnosed with incident cancer of any type or dying of cancer. By triangulating evidence from randomized trials and Mendelian randomization, we have shown that a unit reduction in blood pressure, achieved through pharmacologic intervention or genetic means, is not consistently associated with any cancer type. Although the possibility of an increased risk for lung cancer associated with pharmacologic blood pressure reduction in RCTs data cannot be completely dismissed, the absence of such associations in the Mendelian randomization mitigates the likelihood that this risk is directly attributable to blood pressure–lowering treatments. The results of this study suggest that hypertension or raised blood pressure is unlikely to be a cause of cancer and that blood pressure reduction through antihypertensive medication is safe in the context of cancer risk.

Perspectives **COMPETENCY IN MEDICAL KNOWLEDGE:** In an analysis of randomized data, which included evidence from RCTs and naturally randomized genetic variants, we found no substantial evidence that long-term pharmacologic blood pressure lowering is associated with either an increased or a decreased risk for overall incident cancer, cancer-related mortality, or selected site-specific cancers, including breast, colorectal, kidney, prostate, and skin cancers. Clinicians can consider long-term blood pressure–lowering treatment to reduce the risk for cardiovascular disease without concern about its impact on cancer and can reassure patients that it is not linked to increased cancer risk.

**TRANSLATIONAL OUTLOOK:** The “class-specific” effect of each antihypertensive drug on the risk for different “site-specific” cancers and their subtypes requires further investigation. Although conducting a RCT to test such a hypothesis may be costly and potentially unfeasible, drug-target Mendelian randomization could represent the most viable alternative to provide randomized evidence in this context.

## Funding Support and Author Disclosures

This research was funded by the British Heart Foundation (PG/18/65/33872 and FS/IPBSRF/22/27060). Dr Rahimi has received grants outside the submitted work from the British Heart Foundation, the Horizon Europe AI4HF consortium (grant R79992/CN001), the Novo Nordisk Oxford Big Data Partnership, the University of Oxford, and UK Research and Innovation’s Global Challenge Research Fund (grant ES/P011055/1); has received consulting fees from Medtronic CRDN; has received honoraria or fees from *Heart*, *PLoS Medicine*, AstraZeneca MEA Region, Medscape, and WebMD Medscape UK; and is the editor-in-chief of *Heart*. Dr Canoy has received support from the UK Research and Innovation Medical Research Council (UKRI MRC) (MR/Y010825/1), the Dunhil Medical Trust (ARVHF2402/7), and the National Institute for Health and Care Research (NIHR) (NIR203982) outside of the submitted work; the views expressed are not necessarily those of these funders; he has also received an honorarium as Specialty Chief Editor of *Frontiers in Cardiovascular Medicine* (Cardiovascular Epidemiology and Prevention). Mrs Bidel has received a PhD fellowship from the British Heart Foundation (FS/PhD/25/29632). Dr Nazarzadeh is supported by a research fellowship from the British Heart Foundation (grant FS/IPBSRF/22/27060); has received reimbursement and honoraria from AstraZeneca, Nemysis, and Albus Health outside the submitted work; and is the statistical adviser for *Heart*. Dr Woodward has received personal fees from Amgen, Kyowa Kirin, and Freeline, outside the submitted work. Dr Sundström has ownership in companies providing services to Itrim, Amgen, Janssen, Novo Nordisk, Eli Lilly, Boehringer Ingelheim, Bayer, Pfizer, and AstraZeneca, outside the submitted work. Drs Rahimi and Canoy received grants from the British Heart Foundation during the conduct of the study. Dr Kjeldsen has received lecture honoraria from Emcure, Getz, Glenmark, J.B. Pharma, Merck, Vector-Intas, and Zydus; and has received study committee honoraria from Takeda. Dr Chalmers has received grants from the National Health and Medical Research Council of Australia, outside the submitted work. All other authors have reported that they have no relationships relevant to the contents of this paper to disclose. Where authors are identified as personnel of the International Agency for Research on Cancer/World Health Organization, the authors alone are responsible for the views expressed in this article, and they do not necessarily represent the decisions, policy, or views of the International Agency for Research on Cancer/World Health Organization.

## Appendix

Supplemental Material

Supplemental Excel

## References

[1] Piccirillo J.F., Tierney R.M., Costas I., Grove L., Spitznagel E.L. (2004). Prognostic importance of comorbidity in a hospital-based cancer registry. JAMA.

[2] Copland E., Canoy D., Nazarzadeh M. (2021). Antihypertensive treatment and risk of cancer: an individual participant data meta-analysis. Lancet Oncol.

[3] Seretis A., Cividini S., Markozannes G. (2019). Association between blood pressure and risk of cancer development: a systematic review and meta-analysis of observational studies. Sci Rep.

[4] Christakoudi S., Kakourou A., Markozannes G. (2020). Blood pressure and risk of cancer in the European Prospective Investigation Into Cancer and Nutrition. Int J Cancer.

[5] Hidayat K., Du X., Zou S.Y., Shi B.M. (2017). Blood pressure and kidney cancer risk: meta-analysis of prospective studies. J Hypertens.

[6] Stocks T., Lukanova A., Bjørge T. (2011). Metabolic factors and the risk of colorectal cancer in 580,000 men and women in the metabolic syndrome and cancer project (Me-Can). Cancer.

[7] Han H., Guo W., Shi W. (2017). Hypertension and breast cancer risk: a systematic review and meta-analysis. Sci Rep.

[8] Scelo G., Larose T.L. (2018). Epidemiology and risk factors for kidney cancer. J Clin Oncol.

[9] Hiam-Galvez K.J., Allen B.M., Spitzer M.H. (2021). Systemic immunity in cancer. Nat Rev Cancer.

[10] Solak Y., Afsar B., Vaziri N.D. (2016). Hypertension as an autoimmune and inflammatory disease. Hypertens Res.

[11] Battistoni A., Tocci G., Coluccia R., Burnier M., Ruilope L.M., Volpe M. (2020). Antihypertensive drugs and the risk of cancer: a critical review of available evidence and perspective. J Hypertens.

[12] Lawlor D.A., Tilling K., Davey Smith G. (2016). Triangulation in aetiological epidemiology. Int J Epidemiol.

[13] Davies N.M., Holmes M.V., Davey Smith G. (2018). Reading Mendelian randomization studies: a guide, glossary, and checklist for clinicians. BMJ.

[14] Rahimi K., Canoy D., Nazarzadeh M. (2019). Investigating the stratified efficacy and safety of pharmacological blood pressure-lowering: an overall protocol for individual patient-level data meta-analyses of over 300 000 randomized participants in the new phase of the Blood Pressure Lowering Treatment Trialists’ Collaboration (BPLTTC). BMJ Open.

[15] Canoy D., Copland E., Nazarzadeh M. (2022). Antihypertensive drug effects on long-term blood pressure: an individual-level data meta-analysis of randomized clinical trials. Heart.

[16] Blood Pressure Lowering Treatment Trialists’ Collaboration (2021). Pharmacological blood pressure lowering for primary and secondary prevention of cardiovascular disease across different levels of blood pressure: an individual participant-level data meta-analysis. Lancet.

[17] Hommel G. (1988). A stagewise rejective multiple test procedure based on a modified Bonferroni test. Biometrika.

[18] Wang R., Lagakos S.W., Ware J.H., Hunter D.J., Drazen J.M. (2007). Statistics in medicine—reporting of subgroup analyses in clinical trials. N Engl J Med.

[19] European Medicines Agency (2017). Multiplicity issues in clinical trials—scientific guideline. https://www.ema.europa.eu/en/multiplicity-issues-clinical-trials-scientific-guideline.

[20] Smith G.D., Ebrahim S. (2003). “Mendelian randomization”: can genetic epidemiology contribute to understanding environmental determinants of disease?. Int J Epidemiol.

[21] Evangelou E., Warren H.R., Mosen-Ansorena D. (2018). Genetic analysis of over 1 million people identifies 535 new loci associated with blood pressure traits. Nat Genet.

[22] Fortier I., Raina P., Van den Heuvel E.R. (2017). Maelstrom Research guidelines for rigorous retrospective data harmonization. Int J Epidemiol.

[23] Hartwig F.P., Davies N.M., Hemani G., Davey Smith G. (2016). Two-sample Mendelian randomization: avoiding the downsides of a powerful, widely applicable but potentially fallible technique. Int J Epidemiol.

[24] Hartwig F.P., Davey Smith G., Bowden J. (2017). Robust inference in summary data Mendelian randomization via the zero modal pleiotropy assumption. Int J Epidemiol.

[25] Hemani G., Bowden J., Davey Smith G. (2018). Evaluating the potential role of pleiotropy in Mendelian randomization studies. Hum Mol Genet.

[26] Bowden J., Davey Smith G., Haycock P.C., Burgess S. (2016). Consistent estimation in Mendelian randomization with some invalid instruments using a weighted median estimator. Genet Epidemiol.

[27] Verbanck M., Chen C.-Y., Neale B., Do R. (2018). Detection of widespread horizontal pleiotropy in causal relationships inferred from Mendelian randomization between complex traits and diseases. Nat Genet.

[28] Bowden J., Davey Smith G., Burgess S. (2015). Mendelian randomization with invalid instruments: effect estimation and bias detection through Egger regression. Int J Epidemiol.

[29] Zhao Q., Wang J., Hemani G., Bowden J., Small D.S. (2020). Statistical inference in two-sample summary-data Mendelian randomization using robust adjusted profile score. Ann Stat.

[30] Qi G., Chatterjee N. (2019). Mendelian randomization analysis using mixture models for robust and efficient estimation of causal effects. Nat Commun.

[31] Burgess S., Bowden J., Fall T., Ingelsson E., Thompson S.G. (2017). Sensitivity analyses for robust causal inference from Mendelian randomization analyses with multiple genetic variants. Epidemiology.

[32] Weikert S., Boeing H., Pischon T. (2008). Blood pressure and risk of renal cell carcinoma in the European prospective investigation into cancer and nutrition. Am J Epidemiol.

[33] Corrao G., Scotti L., Bagnardi V., Sega R. (2007). Hypertension, antihypertensive therapy and renal-cell cancer: a meta-analysis. Curr Drug Saf.

[34] Grossman E., Messerli F.H., Boyko V., Goldbourt U. (2002). Is there an association between hypertension and cancer mortality?. Am J Med.

[35] Petrelli F., Ghidini A., Cabiddu M. (2021). Effects of hypertension on cancer survival: a meta-analysis. Eur J Clin Invest.

[36] Harding J.L., Sooriyakumaran M., Anstey K.J. (2016). Hypertension, antihypertensive treatment and cancer incidence and mortality: a pooled collaborative analysis of 12 Australian and New Zealand cohorts. J Hypertens.

[37] Clarke R., Wright N., Walters R. (2023). Genetically predicted differences in systolic blood pressure and risk of cardiovascular and noncardiovascular diseases: a Mendelian randomization study in Chinese adults. Hypertension.

[38] Chan I.I., Kwok M.K., Schooling C.M. (2021). Blood pressure and risk of cancer: a Mendelian randomization study. BMC Cancer.

[39] Alcala K., Mariosa D., Smith-Byrne K. (2022). The relationship between blood pressure and risk of renal cell carcinoma. Int J Epidemiol.

[40] Bangalore S., Kumar S., Kjeldsen S.E. (2011). Antihypertensive drugs and risk of cancer: network meta-analyses and trial sequential analyses of 324,168 participants from randomized trials. Lancet Oncol.

[41] Sipahi I. (2022). Risk of cancer with angiotensin-receptor blockers increases with increasing cumulative exposure: meta-regression analysis of randomized trials. PLoS One.

[42] Sipahi I., Debanne S.M., Rowland D.Y., Simon D.I., Fang J.C. (2010). Angiotensin-receptor blockade and risk of cancer: meta-analysis of randomized controlled trials. Lancet Oncol.

[43] Weiss W. (1997). Cigarette smoking and lung cancer trends. A light at the end of the tunnel?. Chest.

[44] Ference B.A., Ray K.K., Catapano A.L. (2019). Mendelian randomization study of ACLY and cardiovascular disease. N Engl J Med.

[45] Gago-Dominguez M., Castelao J.E., Yuan J.-M., Ross R.K., Yu M.C. (2002). Lipid peroxidation: a novel and unifying concept of the etiology of renal cell carcinoma (United States). Cancer Causes Control.

[46] George A.J., Thomas W.G., Hannan R.D. (2010). The renin–angiotensin system and cancer: old dog, new tricks. Nat Rev Cancer.

[47] Grant K., Loizidou M., Taylor I. (2003). Endothelin-1: a multifunctional molecule in cancer. Br J Cancer.

[48] Rahimi K., Emberson J., McGale P. (2011). Effect of statins on atrial fibrillation: collaborative meta-analysis of published and unpublished evidence from randomized controlled trials. BMJ.

[49] Pogue J., Walter S.D., Yusuf S. (2009). Evaluating the benefit of event adjudication of cardiovascular outcomes in large simple RCTs. Clin Trials.

[50] Yarmolinsky J., Díez-Obrero V., Richardson T.G. (2022). Genetically proxied therapeutic inhibition of antihypertensive drug targets and risk of common cancers: A mendelian randomization analysis. PLoS Med.

