# Supplementary material for: Blood Pressure Lowering and Risk of Cancer: Individual Participant-Level Data Meta-Analysis and Mendelian Randomization Studies
Source: JACC CardioOncol. 2025 May 13;7(5):609–23. doi: 10.1016/j.jaccao.2025.03.005 (PMC7618006; doi:10.1016/j.jaccao.2025.03.005)
Supplement: Supplemental Material [file mmc1.docx]

**Supplemental Material**

**Blood pressure-lowering and risk of cancer: individual participant-level data meta-analysis and Mendelian randomization studies**

Milad Nazarzadeh, DPhil; ^1,2*^ Emma Copland, MSc; ^1-3*^ Karl Smith Byrne, DPhil; ^4^ Dexter Canoy, MD; ^5^ Zeinab Bidel, MSc; ^1,2^ Prof Mark Woodward, PhD; ^6,7^ Qianqian Yang, MSc;^1,2^ James McKay, PhD; ^8^ Anders Mälarstig, PhD; ^9,10^ Åsa Hedman, PhD; ^9,10^ Prof John Chalmers, MD; ^6^ Prof Koon K. Teo, MD; ^11^ Prof Carl J. Pepine, MD; ^12^ Prof Barry R. Davis, MD;^13^ Prof Sverre Kjeldsen, MD; ^14^, Prof Johan Sundström, MD; ^15^, Prof Kazem Rahimi, FRCP; ^1-3^, On behalf of the Blood Pressure Lowering Treatment Trialists’ Collaboration

^* Equal contribution^

^1^ Deep Medicine, Oxford Martin School, University of Oxford, Oxford, UK

^2^ Nuffield Department of Women’s and Reproductive Health, University of Oxford, Oxford, UK

^3^ NIHR Oxford Biomedical Research Centre, Oxford University Hospitals NHS Foundation Trust, Oxford, UK

﻿^4^ Cancer Epidemiology Unit, Nuffield Department of Population Health, University of Oxford, UK

^5^ Population Health Sciences Institute, Newcastle University, Newcastle, UK

^6^ The George Institute for Global Health, University of New South Wales, Sydney, Australia

^7^ The George Institute for Global Health, School of Public Health, Imperial College London, London, UK

^8^ Genomic Epidemiology Branch, International Agency for Research on Cancer (IARC-WHO), Lyon, France

^9^ External Science and Innovation, Pfizer Worldwide Research, Development and Medical, Stockholm, Sweden

^10^ Department of Medicine, Karolinska Institute, Stockholm, Sweden

^11^ Population Health Research Institute, Hamilton Health Sciences, McMaster University, Hamilton, Ontario, Canada

^12^ College of Medicine, University of Florida, Gainesville, Florida, USA

^13^ The University of Texas School of Public Health, Houston, Texas, USA

^14^ Department of Cardiology, University of Oslo, Ullevaal Hospital, Oslo, Norway

^15^ Department of Medical Sciences, Clinical Epidemiology, Uppsala University, Uppsala, Sweden

**Correspondence to**

Prof Kazem Rahimi, Nuffield Department of Women’s and Reproductive Health, University of Oxford, Oxford, UK

Address: Deep Medicine group, second floor, Osney One Building, Osney Mead, Oxfordshire, Oxford, United Kingdom; post code: OX2 0EW

Email: [kazem.rahimi@wrh.ox.ac.uk](mailto:kazem.rahimi@wrh.ox.ac.uk)

T: +44 1865 617 201

#

# Supplementary Method 1. Details of the design of the individual participant data meta-analysis.

**Individual participant data meta-analysis designs**

There are two main types of individual participant data (IPD) meta-analysis designs: one-stage and two-stage. The “two-stage” IPD meta-analysis is similar to the conventional meta-analysis of summary statistics, but instead of extracting summary statistics from published papers, researchers acquire individual-level data and then estimate treatment effects (e.g., hazard ratios, mean differences, etc) for each trial separately, based on a “unified statistical analysis plan” across all trials. All other details are identical to classic meta-analysis methods. On the other hand, the “one-stage” IPD meta-analysis is an approach that combines data from all “individual participants” across trials into a single comprehensive statistical model. Unlike the two-stage approach, where trials are analyzed independently, the one-stage method analyzes all data simultaneously while accounting for the clustering of participants within trials. This approach uses advanced statistical techniques, such as mixed-effects or stratified models, to allow each trial to have its own baseline hazard function or intercept while estimating a common treatment effect across all trials. The one-stage method offers greater precision and statistical power by leveraging the full variability of individual-level data. It is particularly advantageous for investigating treatment-covariate interactions, effect modification and subgroup analysis, as it can incorporate both individual- and trial-level covariates into the same model. In the context of the current study that focuses on cancer, it should be noted that the one-stage approach has the added advantage of enabling time-stratified analyses (**Figure 2**) and assessing heterogeneity of effects by baseline variables (**Figure 3**). Such subgroup analyses are not straightforward with the two-stage IPD method. The table below compares one-stage, two-stage, and conventional meta-analysis methods.

| Comparison of one-stage, two-stage, and conventional meta-analysis methods | | | |
| --- | --- | --- | --- |
| **Aspect** | **One-Stage Meta-Analysis** | **Two-Stage Meta-Analysis** | **Conventional Meta-Analysis** |
| **Definition** | Combines individual-level data from all trials into a single statistical model. | Analyses each trial separately to calculate trial-specific estimates, which are then pooled using an aggregate data meta-analysis method. | Combines summary-level data (e.g., effect sizes, confidence intervals) reported in published trials. |
| **Data Requirement** | Requires individual-level participant data from all trials. | Requires individual-level participant data from all trials. | Requires only published or aggregate summary data from trials. |
| **Analysis unit** | Each participant | Trial | Trial |
| **Statistical Power** | Greater statistical power as all data is used simultaneously, leveraging individual-level variability. | Relatively lower power due to independent analyses of trials before pooling. | Relatively lower power due to reliance on summary data. |
| **Precision of Estimates** | Provides more precise estimates by using the full individual information | May yield less precise pooled estimates, particularly with small sample sizes or few trials. | Limited precision as it depends on the quality and consistency of published summary data. |
| **Handling of Covariates** | Facilitates exploration of complex covariate interactions and effect modification at both trial and individual levels. | Limited ability to model covariate interactions or effect modification across studies. | Does not allow for covariate-level exploration or individual-level adjustments. |
| **Flexibility** | Can incorporate complex models (e.g., effect modification, stratification, subgroup analysis, time-dependent covariates). | Simpler and more intuitive but less flexible for complex analyses. | Very limited flexibility; focuses on overall effect estimates. |
| **Heterogeneity** | Allows direct modelling heterogeneity within the framework of the model. | Provides measures of aggregate level heterogeneity (e.g., I², τ²) and may overlook nuanced patterns. | Assesses heterogeneity using summary statistics but lacks granularity. |
| **Ease of Implementation** | Requires advanced statistical expertise and computational resources. | Simpler to implement using established meta-analysis software. | Easiest to implement using standard tools (e.g., RevMan, Excel). |

**Stratified Cox proportional hazard model**

In the one-stage IPD approach, individual-level data from different trials, which include participants with varying baseline risks and characteristics, are pooled together and treated as a single dataset for modelling purposes (similar to a large multicentre trial). This requires careful consideration to ensure appropriate statistical analysis. Consequently, accounting for the clustering of participants across trials is an important methodological consideration.^1-3^ We used stratified Cox proportional hazards models to pool hazard ratios (HRs) across trials, treating each trial as a stratum in the model to account for trial-specific baseline hazard variations. This approach ensures that between-trial heterogeneity does not affect effect estimation. A stratified Cox model is a variation of the Cox proportional hazards regression model that accounts for stratification factors by allowing the baseline hazard function to vary across levels of a stratifying variable while keeping the regression coefficients constant across those strata. This approach is particularly useful when there are clusters within the data (e.g., trials, centres, or specific cohorts) that have different baseline hazard functions. ^4-8^

Furthermore, in blood pressure -lowering trials, the primary source of heterogeneity is the variability in the blood pressure reduction achieved in each trial, which is mainly influenced by study design and trial intervention type. Therefore, it is reasonable to assume that the only source of heterogeneity is the magnitude of blood pressure reduction across trials. As such, we used a fixed-effects Cox model, adjusted for blood pressure reduction across trials, to address this known source of heterogeneity.^4-10^

**Standardization of the effect sizes**

Standardization of effect sizes is appropriate when the objective is to pool the effects of blood pressure (BP)-lowering treatments and express the effect for a fixed level of BP reduction. This approach is essential for such analyses because the magnitude of BP reduction varies across trials. Standardization facilitates adjustment for this heterogeneity by assigning greater weight to trials with larger BP reductions, particularly when the hypothesis focuses on assessing the BP-mediated effect. In practical terms, this implies that all else being equal, trials with minimal BP reductions between treatment arms are assigned proportionately lower weights than they would be in the absence of standardization.^9^

Another additional advantage of standardization is that it permits the inclusion of a wide range of BP-lowering trials without necessitating an arbitrary threshold for trial-level achieved BP reduction. For instance, in head-to-head trials comparing one drug with another, the achieved reduction is often modest. Rather than excluding these trials (e.g., with 1 or 2 mmHg BP reduction), standardization enables their inclusion in the analysis, thereby enhancing statistical power while assigning lower weights to account for the smaller achieved BP reduction. To estimate the effect size of treatment per a fixed amount of BP reduction, it is therefore essential to standardize effect sizes to a predefined and clinically meaningful BP level. Without this standardization, the estimated effect size (e.g., hazard ratio) would lack scale, and the interpretation of relative risk reduction would have unclear clinical implications.

For standardization, first, for each trial, the average achieved BP reduction was estimated using a linear mixed-effects model, with further methodological details reported elsewhere.^15^ This value (i.e., achieved BP reduction) was subsequently treated as the trial-level BP reduction for each trial participant and incorporated into the dataset as a new variable for analysis. A stratified Cox model was then fitted to estimate the hazard ratio for the treatment effect, adjusted for this trial-level BP reduction. Finally, the relative effect size was rescaled to correspond to a 5 mmHg reduction in systolic BP and a 3 mmHg reduction in diastolic BP, respectively.^7,8^

# Supplementary Method 2. Summary of Genome-Wide Association Studies used for SNP-outcome association.

**Breast cancer**:

The breast cancer genome-wide association study (GWAS) involved participation from seventy-eight studies as part of the OncoArray consortium. Sixty-seven of these studies contributed data on European ancestry, while twelve provided data on Asian ancestry, excluding one Norwegian study due to the absence of controls. The research predominantly consisted of population-based case-control studies or case-control studies nested within population-based cohorts, with some studies specifically targeting cases with a familial history of breast cancer. All participating studies submitted essential information on disease status, age at diagnosis or observation, with many also providing detailed clinico-pathological and lifestyle data. This wealth of information has been carefully curated into the Breast Cancer Association Consortium database. The quality control (QC) stage for the GWAS involved detailed protocols to ensure data integrity. This process included genotype calling, handling of potential errors, and consistency checks across datasets. The GWAS included 122,977 cases and 105,974 controls of European ancestry, and 14,068 cases and 13,104 controls of East Asian ancestry.^11^

**Colorectal cancer**

The colorectal cancer GWAS included data from five primary GWAS alongside a meta-analysis with ten published GWAS, comprising 34,627 cases and 71,379 controls of European ancestry. The analysis utilised rigorous QC measures and imputation based on the 1000 Genomes and UK10K data as reference. After excluding variants with minor allele frequency less than 0.5% and imputation quality score below 0.8, associations between colorectal cancer status and single nucleotide polymorphism (SNP) genotypes were assessed using logistic regression, with risk estimates combined through inverse-variance weighted fixed-effects meta-analysis.^12^

**Kidney cancer**

The kidney cancer GWAS incorporated data from six genome-wide scans, including two new and four previous studies, totaling 10,784 cases and 20,406 controls of European ancestry. Quality control measures were rigorously applied to ensure data integrity. The statistical analysis utilized fixed-effects meta-analysis for discovery and targeted replication in an independent set of 3,182 cases and 6,301 controls. This approach confirmed six known renal cell carcinoma (RCC) risk loci and identified seven new loci, enhancing understanding of RCC's genetic basis.^13^

**Lung cancer**

The study involved a large-scale analysis including 14,803 cases and 12,262 controls of European descent, with subsequent imputation and logistic regression analysis to explore the associations between genetic variants and lung cancer risk. The study successfully identified 18 new susceptibility loci, highlighting the heterogeneity in genetic risk across lung cancer subtypes and emphasizing the complexity of its genetic underpinnings.^14^

**Prostate cancer**

The prostate cancer GWAS study assembled a new sample series from 52 studies for genotyping with the OncoArray, after rigorous QC, yielding 46,939 prostate cancer cases and 27,910 controls of European ancestry for analysis. Genotypes were imputed to the 1000 Genomes Project reference panel, and a fixed-effects meta-analysis was performed, including 79,194 prostate cancer cases and 61,112 controls. The study identified 63 novel prostate cancer susceptibility variants through logistic regression analysis, adjusted for principal components and study-specific covariates, highlighting significant advancements in understanding prostate cancer genetics.^15^

**Skin cancer**

The basal cell carcinoma (BCC) study consisted of a two-stage GWAS meta-analysis, totaling 17,187 cases and 287,054 controls. Stage 1 analysis involved 12,945 self-reported BCC cases and 274,252 controls of European ancestry from 23andMe research participants. Stage 2 comprised an independent GWAS cohort of 4,242 BCC cases and 12,802 controls of European ancestry from the Nurses’ Health Study and Health Professionals Follow-Up Study. QC measures and genotype imputation were rigorously applied, with meta-analysis combining stages 1 and 2 results to identify 14 novel susceptibility loci for BCC.^16^ The cutaneous squamous cell carcinoma (SCC) GWAS study pooled data from six international cohorts, totaling 19,149 SCC cases and 680,049 controls. This comprehensive analysis confirmed 14 previously associated loci and identified eight new susceptibility loci. The study leveraged strict quality control measures and sophisticated genotyping and imputation strategies to ensure data integrity and accuracy. The statistical approach involved logistic regression adjusted for principal components to account for population stratification, with meta-analysis techniques applied to aggregate findings across cohorts.^17^ The cutaneous melanoma GWAS meta-analysis involved 36,760 cases and 375,188 controls, identifying 54 significant loci with 68 independent SNPs. The study highlighted the importance of nevogenesis, pigmentation, and telomere maintenance in melanoma susceptibility, revealing new pathways for research. The analysis also integrated GWAS data of nevus count and hair color, uncovering 31 potential secondary loci, bringing the total to 85 susceptibility loci. This extensive study provides new insights into the genetic architecture of cutaneous melanoma.^18^

Supplementary Method 3. Summary of randomized clinical trials included in individual participant data meta-analysis

| **Trial** | **Country** | **Type of trial** | **Randomization groups** | | **Trial sample size (% women)** | **Age**  **[mean, SD]** | **Percentage of current smokers (%, n)** | **Body mass index (kg/m^2^)**  **[mean, SD]** | **Median follow-up duration (year)** | **Explicit exclusion of cancer patients at baseline** | **Source of cancer outcome** | **Adjudication** | **Level of detail of cancer outcomes provided** |
| --- | --- | --- | --- | --- | --- | --- | --- | --- | --- | --- | --- | --- | --- |
|  |  |  | Treatment | Comparator |  |  |  |  |  |  |  |  |  |
| AASK*^19^ | USA | Intensive | More intense BP-lowering | Less intense BP-lowering | 1094 (39) | 54 (11) | 29 (321) | 30.6 (6.6) | 4.8 | No | Routine adverse event | No | Site of cancer diagnosis (lung, colon, breast, prostate, skin, other types) |
| ABCD*^20^ | USA | Intensive | More intense BP-lowering | Less intense BP-lowering | 950 (33) | 58 (8) | 14 (128) | 31.7 (5.7) | 4.7 | Yes (patients with active cancer) | Routine adverse event | No | Cancer diagnosis yes/no (no information on site of cancer) |
| ACCORD^21^ | USA and Canada | Intensive | More intense BP-lowering | Less intense BP-lowering | 4733 (48) | 63 (7) | 13 (626) | 32.1 (5.5) | 4.7 | Yes (cancer within 2 years other than non-melanoma skin) | Pre-specified safety outcome | Yes | Fatal cancer yes/no (no information on site of cancer) |
| ACTIVE I^22^ | Multi-country | Placebo-controlled | ARB | Placebo | 9016 (39) | 70 (10) | 22 (9269) | 29.1 (5.8) | 4.1 | No | Routine adverse event | No | Site of cancer diagnosis (lung, colon, breast, prostate, skin, other) |
| ADVANCE^23^ | Multi-country | Placebo-controlled | ACEi and Diuretic | Placebo | 11,140 (43) | 66 (6) | 14 (1550) | 28.3 (5.2) | 4.2 | No | Routine adverse event | No | ICD-10 codes for site of cancer |
| ALLHAT^24^ | Multi-country | Head-to-head | Diuretic | ACEi  CCB  Alpha-blocker | 42,418 (47) | 67 (8) | 22 (9269) | 29.6 (5.9) | 4.8 | No | Pre-specified safety outcome | Yes | Site of cancer diagnosis (lung, colon, breast, prostate, bladder, other) |
| ANBP2^25^ | Australia | Head-to-head | Diuretic | ACEi | 6083 (51) | 73 (5) | 7 (431) | 27.1 (4.2) | 4.1 | No | Pre-specified safety outcome | Yes | ICD-9 codes for site of cancer |
| ASCOT-BPLA^26^ | Multi-country | Head-to-head | CCB-based | Beta-blocker-based | 19,257 (23) | 63 (9) | 33 (6277) | 28.7 (4.6) | 5.3 | No | Routine adverse event | No | Fatal cancer yes/no (no site of cancer) |
| BENEDICT^27^ | Italy | Placebo-controlled | ACEi  CCB  ACEi and CCB | Placebo | 1204 (47) | 62 (8) | 12 (146) | 29.1 (4.7) | 3.1 | Yes | Routine adverse event | No | Site of cancer diagnosis (lung, colon, breast, prostate, other) |
| CAMELOT^28^ | Multi-country | Placebo-controlled | CCB  ACEI | Placebo | 1991 (26) | 58 (10) | 26 (528) | 29.8 (5.3) | 1.6 | No | Routine adverse event | No | Site of cancer diagnosis (text description) |
| CASE-J^29^ | Japan | Head-to-head | CCB | ARB | 4703 (45) | 64 (11) | 22 (1025) | 24.5 (3.7) | 3.1 | Yes (cancer within 5 years of enrolment) | Routine adverse event | No | Site of cancer diagnosis (text description) |
| COLM^30^ | Japan | Head-to-head | Diuretic and ARB | CCB and ARB | 5141 (48) | 74 (5) | 11 (551) | 24.3 (3.4) | 3.0 | Yes (patients with malignant tumours) | Routine adverse event | Yes | MedDRA codes for site of cancer |
| CONVINCE^31^ | Multi-country | Head-to-head | CCB | Beta-blocker Diuretic | 16476 (55) | 66 (7) | 23 (3795) | - | 2.8 | Yes (untreated malignancy within 5 years of enrolment) | Pre-specified safety outcome | Yes | Cancer diagnosis yes/no (no information on site of cancer) |
| COPE^32^ | Japan | Head-to-head | Beta-blocker and CCB Diuretic and CCB | ARB and CCB | 3293 (49) | 64 (11) | 21 (700) | 24.5 (3.4) | 3.6 | Yes (cancer 5 years prior to study entry) | Routine adverse event | No | MedDRA codes for site of cancer |
| DIABHYCAR^33^ | The Nether-lands | Placebo-controlled | ACEi | Placebo | 4912 (30) | 65 (8) | 15 (756) | 29.2 (4.6) | 3.9 | Yes | Routine adverse event | No | Site of cancer diagnosis (text description) |
| Dutch-TIA^34^ | The Nether-lands | Placebo-controlled | Beta-blocker | Placebo | 1473 (36) | 64 (10) | 47 (693) | - | 2.3 | No | Routine adverse event | No | Fatal cancer yes/no (no information on site of cancer) |
| ELSA^35^ | Multi-country | Head-to-head | CCB | Beta-blocker | 2334 (45) | 57 (7) | 20 (478) | 27.2 (3.8) | 3.4 | No | Routine adverse event | No | ICD-9 codes for site of fatal cancer |
| EUROPA^36^ | Multi-country (Europe) | Placebo-controlled | ACEi | Placebo | 12,218 (15) | 61 (9) | 15 (1862) | 27.4 (3.5) | 4.2 | No | Routine adverse event | No | Site of cancer diagnosis (text description) |
| EWPHE^37^ | Multi-country | Placebo-controlled | Diuretic | Placebo | 840 (70) | 71 (8) | 17 (143) | 26.4 (4.5) | 4.6 | Yes | Routine adverse event | No | ICD-8 codes for site of cancer |
| HDFP^38^ | USA | Intensive | More intense BP-lowering | Less intense BP-lowering | 10,940 (46) | 51 (10) | 39 (4248) |  | 6.7 | No | Pre-specified safety outcome | Yes | Site of cancer diagnosis (lung, colon, breast, prostate, bladder, other); ICD-8 codes |
| HIJ-CREATE^39^ | Japan | Head-to-head | ARB | Non-ARB | 2049 (20) | 65 (9) | 25 (509) | 24.6 (3) | 4.0 | Yes (known malignant neoplasm) | Routine adverse event | Yes | Site of cancer diagnosis (text description) |
| HOMED-BP*^40^ | Japan | Intensive | More intense BP-lowering | Less intense BP-lowering | 3518 (50) | 60 (10) | 21 (743) | 24.4 (3.5) | 4.9 | No | Routine adverse event | No | ICD-10 codes for site of fatal cancer |
| HOPE^41^ | Multi-country | Placebo-controlled | ACEi | Placebo | 9297 (27) | 66 (7) | 14 (1319) | 27.7 (4.4) | 4.5 | No | Pre-specified safety outcome | Yes | Cancer diagnosis yes/no (no information on site of cancer) |
| IDNT^42^ | USA | Placebo-controlled | ARB  CCB | Placebo | 1715 (34) | 59 (8) | - | 30.8 (5.8) | 2.6 | No | Routine adverse event | Yes | Site of cancer diagnosis (text description) |
| INVEST^43^ | Multi-country | Head-to-head | CCB | Non-CCB | 21,320 (52) | 66 (10) | 12 (2809) | 29.2 (7.1) | 2.8 | Yes (skin, prostate and other cancer with shortened survival expected) | Pre-specified safety outcome | Yes | Site of cancer diagnosis (lung, colon, breast, prostate, bladder, other) |
| JMIC-B^44^ | Japan | Head-to-head | CCB | ACEi | 1650 (31) | 65 (85) | 34 (563) | 24 (2.9) | 2.3 | No | Pre-specified safety outcome | Yes | ICD-9 codes for site of fatal cancer |
| LIFE^45^ | Multi-country | Head-to-head | ARB | Beta-blocker | 9193 (54) | 67 (7) | 16 (1499) | 28 (4.8) | 4.9 | No | Pre-specified safety outcome | No | Site of cancer diagnosis (text description) |
| MOSES^46^ | Germany and Austria | Head-to-head | CCB | ARB | 1352 (46) | 68 (10) | 18 (247) | 27.5 (4.3) | 3.3 | No | Routine adverse event | Yes | ICD-10 codes for site of cancer |
| NICS-EH^47^ | Japan | Head-to-head | Diuretic | CCB | 414 (67) | 70 (7) | 9 (38) | 23.4 (3.1) | 3.2 | No | Pre-specified safety outcome | Yes | Site of cancer diagnosis (lung, bowel, breast, other) |
| ONTARGET^48^ | Multi-country | Head-to-head | ACEi and ARB | ACEi  ARB | 25,620 (27) | 67 (7) | 13 (3225) | 28.2 (4.8) | 4.8 | No | Pre-specified safety outcome | No | Site of cancer diagnosis (lung, colon, breast, prostate, other) |
| PART-2^49^ | New Zealand | Placebo-controlled | ACEi | Placebo | 617 (18) | 60 (8) | 16 (100) | 26.8 (3.6) | 4.6 | No | Routine adverse event | No | Site of cancer diagnosis (lung, colon, breast, other) |
| PREVEND IT^50^ | The Nether-lands | Placebo-controlled | ACEi | Placebo | 864 (35) | 51 (12) | 40 (345) | 26.4 (4.4) | 3.8 | No | Routine adverse event | No | ICD-10 codes for site of fatal cancer |
| PREVENT^51^ | USA and Canada | Placebo-controlled | CCB | Placebo | 825 (20) | 57 (10) | 25 (204) | 28 (4.8) | 3.0 | No | Pre-specified safety outcome | Yes | Site of cancer diagnosis (text description) |
| PRoFESS^52^ | Multi-country | Placebo-controlled | ARB | Placebo | 19,798 (36) | 66 (8) | 21 (4231) | 26.8 (5) | 2.5 | Yes | Pre-specified safety outcome | Yes | Site of cancer diagnosis (text description) |
| PROGRESS^53^ | Multi-country (Asia, Australa-sia and Europe) | Placebo-controlled | ACEi and/or Diuretic | Placebo | 6105 (30) | 64 (10) | 21 (1279) | 25.7 (3.8) | 3.9 | No | Pre-specified safety outcome | No | Site of cancer diagnosis (lung, colon, breast, prostate, other) |
| SHEP^54^ | USA | Placebo-controlled | Diuretic and Beta-blocker | Placebo | 4736 (57) | 72 (7) | 13 (597) | 27.1 (4.8) | 5.0 | Yes | Routine adverse event | Yes | Cancer diagnosis yes/no (no information on site of cancer) |
| SPRINT^55^ | USA and Puerto Rico | Intensive | More intense BP-lowering | Less intense BP-lowering | 9361 (36) | 68 (9) | 13 (1240) | 29.9 (5.8) | 3.0 | Yes (cancer in past 2 years; exceptions for patients able to complete trial) | Routine adverse event | Yes | MedDRA codes for site of cancer |
| STOP Hyper-tension-2^56^ | Sweden | Head-to-head | Beta-blocker and/or Diuretic | ACEi  CCB | 6614 (67) | 76 (4) | 9 (594) | 26.7 (4) | 4.5 | No | Routine adverse event | No | Site of cancer diagnosis (text description) |
| Syst-Eur^57^ | Multi-country | Placebo-controlled | CCB | Placebo | 4695 (67) | 70 (7) | 7 (343) | 27 (4.1) | 2.6 | No | Pre-specified safety outcome | Yes | Site of cancer diagnosis (lung, colon, breast, prostate, other) |
| TRANSCEND^58^ | Multi-country | Placebo-controlled | ARB | Placebo | 5926 (43) | 68 (7) | 10 (582) | 28.2 (4.8) | 4.9 | No | Pre-specified safety outcome | No | Site of cancer diagnosis (lung, colon, breast, prostate, other) |
| VALIS ^59^ | Japan | Intensive | More intense BP-lowering | Less intense BP-lowering | 3079 (62) | 76 (4) | 15 (450) | 23.5 (3.4) | 2.6 | No | Routine adverse event | Yes | MedDRA codes for site of cancer |
| VALUE^60^ | Multi-country | Head-to-head | CCB-based | ARB-based | 15,245 (42) | 67 (8) | 24 (3664) | 28.6 (5) | 4.2 | No | Routine adverse event | No | MedDRA codes for site of cancer |

* AASK, ABCD and HOMED-BP also included head-to-head drug class comparisons in their study design.

ACEI=angiotensin-converting enzyme inhibitors; ARB=angiotensin-II receptor blockers; CCB=calcium channel blockers.

SD: standard deviation

Supplementary Figure 1. Effects of blood pressure-lowering on incident cancer, cancer death and site-specific cancer, accounting for competing risk of non-cancer death.

SBP: systolic blood pressure, DBP: diastolic blood pressure, HR: hazard ratio, CI: confidence intervals

**Panel A: Systolic blood pressure reduction**


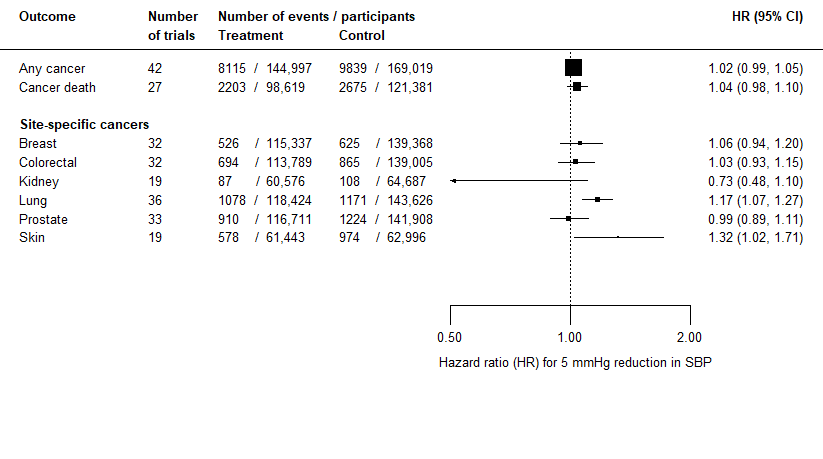


**Panel B: Diastolic blood pressure reduction**


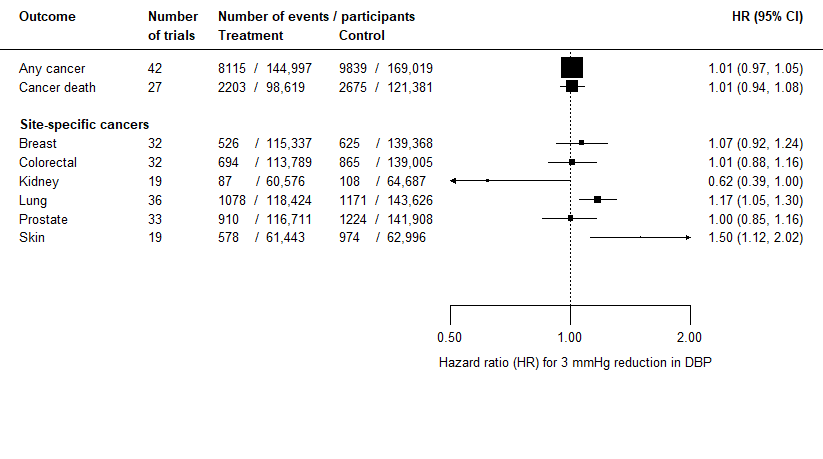


**References**

1. Debray TP, Moons KG, van Valkenhoef G, et al. Get real in individual participant data (IPD) meta-analysis: a review of the methodology. *Res Synth Methods* 2015; **6**(4): 293-309.

2. Bowden J, Tierney JF, Simmonds M, Copas AJ, Higgins JP. Individual patient data meta-analysis of time-to-event outcomes: one-stage versus two-stage approaches for estimating the hazard ratio under a random effects model. *Res Synth Methods* 2011; **2**(3): 150-62.

3. Tudur Smith C, Williamson PR. A comparison of methods for fixed effects meta-analysis of individual patient data with time to event outcomes. *Clin Trials* 2007; **4**(6): 621-30.

4. Bidel Z, Nazarzadeh M, Canoy D, et al. Sex-Specific Effects of Blood Pressure Lowering Pharmacotherapy for the Prevention of Cardiovascular Disease: An Individual Participant-Level Data Meta-Analysis. *Hypertension* 2023; **80**(11): 2293-302.

5. Nazarzadeh M, Bidel Z, Canoy D, et al. Blood pressure-lowering treatment for prevention of major cardiovascular diseases in people with and without type 2 diabetes: an individual participant-level data meta-analysis. *Lancet Diabetes Endocrinol* 2022; **10**(9): 645-54.

6. Nazarzadeh M, Bidel Z, Canoy D, et al. Blood pressure lowering and risk of new-onset type 2 diabetes: an individual participant data meta-analysis. *Lancet* 2021; **398**(10313): 1803-10.

7. Blood Pressure Lowering Treatment Trialists C. Age-stratified and blood-pressure-stratified effects of blood-pressure-lowering pharmacotherapy for the prevention of cardiovascular disease and death: an individual participant-level data meta-analysis. *Lancet* 2021; **398**(10305): 1053-64.

8. Blood Pressure Lowering Treatment Trialists C. Pharmacological blood pressure lowering for primary and secondary prevention of cardiovascular disease across different levels of blood pressure: an individual participant-level data meta-analysis. *Lancet* 2021; **397**(10285): 1625-36.

9. Nazarzadeh M, Canoy D, Bidel Z, et al. The Blood Pressure Lowering Treatment Trialists' Collaboration: methodological clarifications of recent reports. *J Hypertens* 2022; **40**(5): 847-52.

10. Copland E, Canoy D, Nazarzadeh M, et al. Antihypertensive treatment and risk of cancer: an individual participant data meta-analysis. *Lancet Oncol* 2021; **22**(4): 558-70.

11. Michailidou K, Lindstrom S, Dennis J, et al. Association analysis identifies 65 new breast cancer risk loci. *Nature* 2017; **551**(7678): 92-4.

12. Law PJ, Timofeeva M, Fernandez-Rozadilla C, et al. Association analyses identify 31 new risk loci for colorectal cancer susceptibility. *Nat Commun* 2019; **10**(1): 2154.

13. Scelo G, Purdue MP, Brown KM, et al. Genome-wide association study identifies multiple risk loci for renal cell carcinoma. *Nat Commun* 2017; **8**(1): 15724.

14. McKay JD, Hung RJ, Han Y, et al. Large-scale association analysis identifies new lung cancer susceptibility loci and heterogeneity in genetic susceptibility across histological subtypes. *Nat Genet* 2017; **49**(7): 1126-32.

15. Schumacher FR, Al Olama AA, Berndt SI, et al. Association analyses of more than 140,000 men identify 63 new prostate cancer susceptibility loci. *Nat Genet* 2018; **50**(7): 928-36.

16. Chahal HS, Wu W, Ransohoff KJ, et al. Genome-wide association study identifies 14 novel risk alleles associated with basal cell carcinoma. *Nat Commun* 2016; **7**(1): 12510.

17. Sarin KY, Lin Y, Daneshjou R, et al. Genome-wide meta-analysis identifies eight new susceptibility loci for cutaneous squamous cell carcinoma. *Nat Commun* 2020; **11**(1): 820.

18. Landi MT, Bishop DT, MacGregor S, et al. Genome-wide association meta-analyses combining multiple risk phenotypes provide insights into the genetic architecture of cutaneous melanoma susceptibility. *Nat Genet* 2020; **52**(5): 494-504.

19. Appel LJ, Wright JT, Jr., Greene T, et al. Intensive blood-pressure control in hypertensive chronic kidney disease. *N Engl J Med* 2010; **363**(10): 918-29.

20. Schrier RW, Estacio RO, Jeffers B. Appropriate Blood Pressure Control in NIDDM (ABCD) Trial. *Diabetologia* 1996; **39**(12): 1646-54.

21. Cushman WC, Evans GW, Byington RP, et al. Effects of intensive blood-pressure control in type 2 diabetes mellitus. *N Engl J Med* 2010; **362**(17): 1575-85.

22. Yusuf S, Healey JS, Pogue J, et al. Irbesartan in patients with atrial fibrillation. *N Engl J Med* 2011; **364**(10): 928-38.

23. Patel A, MacMahon S, Chalmers J, et al. Effects of a fixed combination of perindopril and indapamide on macrovascular and microvascular outcomes in patients with type 2 diabetes mellitus (the ADVANCE trial): a randomized controlled trial. *Lancet* 2007; **370**(9590): 829-40.

24. Major outcomes in high-risk hypertensive patients randomized to angiotensin-converting enzyme inhibitor or calcium channel blocker vs diuretic: The Antihypertensive and Lipid-Lowering Treatment to Prevent Heart Attack Trial (ALLHAT). *Jama* 2002; **288**(23): 2981-97.

25. Wing LM, Reid CM, Ryan P, et al. A comparison of outcomes with angiotensin-converting--enzyme inhibitors and diuretics for hypertension in the elderly. *N Engl J Med* 2003; **348**(7): 583-92.

26. Dahlöf B, Sever PS, Poulter NR, et al. Prevention of cardiovascular events with an antihypertensive regimen of amlodipine adding perindopril as required versus atenolol adding bendroflumethiazide as required, in the Anglo-Scandinavian Cardiac Outcomes Trial-Blood Pressure Lowering Arm (ASCOT-BPLA): a multicentre randomized controlled trial. *Lancet* 2005; **366**(9489): 895-906.

27. Ruggenenti P, Fassi A, Ilieva AP, et al. Preventing microalbuminuria in type 2 diabetes. *N Engl J Med* 2004; **351**(19): 1941-51.

28. Park S, Yan P, Cerezo C, Jeffers BW. Effect of visit-to-visit blood pressure variability on cardiovascular events in patients with coronary artery disease and well-controlled blood pressure. *J Am Soc Hypertens* 2016; **10**(10): 799-810.

29. Nakao K, Hirata M, Oba K, et al. Role of diabetes and obesity in outcomes of the candesartan antihypertensive survival evaluation in Japan (CASE-J) trial. *Hypertens Res* 2010; **33**(6): 600-6.

30. Ogihara T, Saruta T, Rakugi H, et al. Combinations of olmesartan and a calcium channel blocker or a diuretic in elderly hypertensive patients: a randomized, controlled trial. *J Hypertens* 2014; **32**(10): 2054-63; discussiom 63.

31. Black HR, Elliott WJ, Grandits G, et al. Principal results of the Controlled Onset Verapamil Investigation of Cardiovascular End Points (CONVINCE) trial. *Jama* 2003; **289**(16): 2073-82.

32. Matsuzaki M, Ogihara T, Umemoto S, et al. Prevention of cardiovascular events with calcium channel blocker-based combination therapies in patients with hypertension: a randomized controlled trial. *J Hypertens* 2011; **29**(8): 1649-59.

33. Marre M, Lievre M, Chatellier G, Mann JF, Passa P, Ménard J. Effects of low dose ramipril on cardiovascular and renal outcomes in patients with type 2 diabetes and raised excretion of urinary albumin: randomized, double blind, placebo controlled trial (the DIABHYCAR study). *Bmj* 2004; **328**(7438): 495.

34. Koudstaal PJ, Algra A, Pop GA, Kappelle LJ, van Latum JC, van Gijn J. Risk of cardiac events in atypical transient ischaemic attack or minor stroke. The Dutch TIA Study Group. *Lancet* 1992; **340**(8820): 630-3.

35. Zanchetti A, Bond MG, Hennig M, et al. Calcium antagonist lacidipine slows down progression of asymptomatic carotid atherosclerosis: principal results of the European Lacidipine Study on Atherosclerosis (ELSA), a randomized, double-blind, long-term trial. *Circulation* 2002; **106**(19): 2422-7.

36. Fox KM. Efficacy of perindopril in reduction of cardiovascular events among patients with stable coronary artery disease: randomized, double-blind, placebo-controlled, multicentre trial (the EUROPA study). *Lancet* 2003; **362**(9386): 782-8.

37. Amery A, Birkenhäger W, Brixko P, et al. Mortality and morbidity results from the European Working Party on High Blood Pressure in the Elderly trial. *Lancet* 1985; **1**(8442): 1349-54.

38. Five-year findings of the hypertension detection and follow-up program. I. Reduction in mortality of persons with high blood pressure, including mild hypertension. Hypertension Detection and Follow-up Program Cooperative Group. *Jama* 1979; **242**(23): 2562-71.

39. Kasanuki H, Hagiwara N, Hosoda S, et al. Angiotensin II receptor blocker-based vs. non-angiotensin II receptor blocker-based therapy in patients with angiographically documented coronary artery disease and hypertension: the Heart Institute of Japan Candesartan Randomized Trial for Evaluation in Coronary Artery Disease (HIJ-CREATE). *Eur Heart J* 2009; **30**(10): 1203-12.

40. Asayama K, Ohkubo T, Metoki H, et al. Cardiovascular outcomes in the first trial of antihypertensive therapy guided by self-measured home blood pressure. *Hypertension Research* 2012; **35**(11): 1102-10.

41. Yusuf S, Sleight P, Pogue J, Bosch J, Davies R, Dagenais G. Effects of an angiotensin-converting-enzyme inhibitor, ramipril, on cardiovascular events in high-risk patients. *N Engl J Med* 2000; **342**(3): 145-53.

42. Lewis EJ, Hunsicker LG, Clarke WR, et al. Renoprotective effect of the angiotensin-receptor antagonist irbesartan in patients with nephropathy due to type 2 diabetes. *N Engl J Med* 2001; **345**(12): 851-60.

43. Pepine CJ, Handberg EM, Cooper-DeHoff RM, et al. A Calcium Antagonist vs a Non–Calcium Antagonist Hypertension Treatment Strategy for Patients With Coronary Artery DiseaseThe International Verapamil-Trandolapril Study (INVEST): A Randomized Controlled Trial. *JAMA* 2003; **290**(21): 2805-16.

44. Yui Y, Sumiyoshi T, Kodama K, et al. Comparison of nifedipine retard with angiotensin converting enzyme inhibitors in Japanese hypertensive patients with coronary artery disease: the Japan Multicenter Investigation for Cardiovascular Diseases-B (JMIC-B) randomized trial. *Hypertens Res* 2004; **27**(3): 181-91.

45. Lindholm LH, Ibsen H, Dahlöf B, et al. Cardiovascular morbidity and mortality in patients with diabetes in the Losartan Intervention For Endpoint reduction in hypertension study (LIFE): a randomized trial against atenolol. *Lancet* 2002; **359**(9311): 1004-10.

46. Schrader J, Lüders S, Kulschewski A, et al. Morbidity and Mortality After Stroke, Eprosartan Compared with Nitrendipine for Secondary Prevention: principal results of a prospective randomized controlled study (MOSES). *Stroke* 2005; **36**(6): 1218-26.

47. Randomized double-blind comparison of a calcium antagonist and a diuretic in elderly hypertensives. National Intervention Cooperative Study in Elderly Hypertensives Study Group. *Hypertension* 1999; **34**(5): 1129-33.

48. Yusuf S, Teo KK, Pogue J, et al. Telmisartan, ramipril, or both in patients at high risk for vascular events. *N Engl J Med* 2008; **358**(15): 1547-59.

49. MacMahon S, Sharpe N, Gamble G, et al. Randomized, placebo-controlled trial of the angiotensin-converting enzyme inhibitor, ramipril, in patients with coronary or other occlusive arterial disease. PART-2 Collaborative Research Group. Prevention of Atherosclerosis with Ramipril. *J Am Coll Cardiol* 2000; **36**(2): 438-43.

50. Asselbergs FW, Diercks GF, Hillege HL, et al. Effects of fosinopril and pravastatin on cardiovascular events in subjects with microalbuminuria. *Circulation* 2004; **110**(18): 2809-16.

51. Pitt B, Byington RP, Furberg CD, et al. Effect of amlodipine on the progression of atherosclerosis and the occurrence of clinical events. PREVENT Investigators. *Circulation* 2000; **102**(13): 1503-10.

52. Sacco RL, Diener HC, Yusuf S, et al. Aspirin and extended-release dipyridamole versus clopidogrel for recurrent stroke. *N Engl J Med* 2008; **359**(12): 1238-51.

53. Randomized trial of a perindopril-based blood-pressure-lowering regimen among 6,105 individuals with previous stroke or transient ischaemic attack. *Lancet* 2001; **358**(9287): 1033-41.

54. Ogihara T, Nakao K, Fukui T, et al. Effects of candesartan compared with amlodipine in hypertensive patients with high cardiovascular risks: candesartan antihypertensive survival evaluation in Japan trial. *Hypertension* 2008; **51**(2): 393-8.

55. A Randomized Trial of Intensive versus Standard Blood-Pressure Control. *New England Journal of Medicine* 2015; **373**(22): 2103-16.

56. Hansson L, Lindholm LH, Ekbom T, et al. Randomized trial of old and new antihypertensive drugs in elderly patients: cardiovascular mortality and morbidity the Swedish Trial in Old Patients with Hypertension-2 study. *Lancet* 1999; **354**(9192): 1751-6.

57. Staessen JA, Fagard R, Thijs L, et al. Randomized double-blind comparison of placebo and active treatment for older patients with isolated systolic hypertension. The Systolic Hypertension in Europe (Syst-Eur) Trial Investigators. *Lancet* 1997; **350**(9080): 757-64.

58. Yusuf S, Teo K, Anderson C, et al. Effects of the angiotensin-receptor blocker telmisartan on cardiovascular events in high-risk patients intolerant to angiotensin-converting enzyme inhibitors: a randomized controlled trial. *Lancet* 2008; **372**(9644): 1174-83.

59. Ogihara T, Saruta T, Rakugi H, et al. Target blood pressure for treatment of isolated systolic hypertension in the elderly: valsartan in elderly isolated systolic hypertension study. *Hypertension* 2010; **56**(2): 196-202.

60. Julius S, Kjeldsen SE, Weber M, et al. Outcomes in hypertensive patients at high cardiovascular risk treated with regimens based on valsartan or amlodipine: the VALUE randomized trial. *Lancet* 2004; **363**(9426): 2022-31.
